# Artificially Induced Epithelial-Mesenchymal Transition in Surgical Subjects: Its Implications in Clinical and Basic Cancer Research

**DOI:** 10.1371/journal.pone.0018196

**Published:** 2011-04-21

**Authors:** Kazuhiko Aoyagi, Keiko Minashi, Hiroyasu Igaki, Yuji Tachimori, Takao Nishimura, Norikazu Hokamura, Akio Ashida, Hiroyuki Daiko, Atsushi Ochiai, Manabu Muto, Atsushi Ohtsu, Teruhiko Yoshida, Hiroki Sasaki

**Affiliations:** 1 Division of Integrative Omics and Bioinformatics, National Cancer Center Research Institute, Chuo-ku, Tokyo, Japan; 2 Division of Genetics, National Cancer Center Research Institute, Chuo-ku, Tokyo, Japan; 3 Department of Surgery, National Cancer Center Hospital, Chuo-ku, Tokyo, Japan; 4 Department of Surgery, National Cancer Center Hospital East, Kashiwa, Chiba, Japan; 5 Department of Pathology, National Cancer Center Hospital East, Kashiwa, Chiba, Japan; 6 Department of Endoscopy, National Cancer Center Hospital East, Kashiwa, Chiba, Japan; 7 Department of Gastroenterology and Hepatology, Graduate School of Medicine, Kyoto University, Sakyo-ku, Kyoto, Japan; The University of Hong Kong, Hong Kong

## Abstract

**Background:**

Surgical samples have long been used as important subjects for cancer research. In accordance with an increase of neoadjuvant therapy, biopsy samples have recently become imperative for cancer transcriptome. On the other hand, both biopsy and surgical samples are available for expression profiling for predicting clinical outcome by adjuvant therapy; however, it is still unclear whether surgical sample expression profiles are useful for prediction via biopsy samples, because little has been done about comparative gene expression profiling between the two kinds of samples.

**Methodology and Findings:**

A total of 166 samples (77 biopsy and 89 surgical) of normal and malignant lesions of the esophagus were analyzed by microarrays. Gene expression profiles were compared between biopsy and surgical samples. Artificially induced epithelial-mesenchymal transition (aiEMT) was found in the surgical samples, and also occurred in mouse esophageal epithelial cell layers under an ischemic condition. Identification of clinically significant subgroups was thought to be disrupted by the disorder of the expression profile through this aiEMT.

**Conclusion and Significance:**

This study will evoke the fundamental misinterpretation including underestimation of the prognostic evaluation power of markers by overestimation of EMT in past cancer research, and will furnish some advice for the near future as follows: 1) Understanding how long the tissues were under an ischemic condition. 2) Prevalence of biopsy samples for *in vivo* expression profiling with low biases on basic and clinical research. 3) Checking cancer cell contents and normal- or necrotic-tissue contamination in biopsy samples for prevalence.

## Introduction

Cancer is a major cause of human deaths in many countries. Gene expression profiles from DNA microarrays are individualized and useful in the diagnosis and prognosis of diseases [1]. Although some artificial factors such as ischemia, hypoxia, hyponutrition, and cold stress possibly occur during surgical resection and sample transportation (Figure S1), surgical samples have long been used as important subjects for clinical and basic cancer research. In accordance with an increase of neoadjuvant therapy (in head and neck, esophageal, lung, pancreatic, prostate, and breast cancers), biopsy samples have recently become imperative for cancer transcriptome. On the other hand, both biopsy and surgical samples are available for expression profiling for predicting clinical outcome by adjuvant therapy (in stomach, colon, liver, bladder, pancreatic, brain, kidney, ovarian, cervical, and breast cancers). The targets for microarray analysis were, for the last ten years, mostly surgical samplesfrom the development and prevalence of two types of microarray: oligonucleotide [2], [ 3] and cDNA [4], [ 5]. However, whether a huge number of accumulated surgical sample expression profiles are useful for prediction by the use of biopsy samples from pretreated patients is still unclear, because little has been done about comparative gene expression profiling between the two kinds of samples.

Chemoradiotherapy (CRT) followed by surgery is the standard therapy for esophageal cancer in Western countries. In Japan, neoadjuvant chemotherapy followed by surgery and definitive CRT are the standard therapies [6], and for locally advanced esophageal cancers (Stage II or III), surgery was the standard therapy there approximately 5 years ago [7]. This enables us to obtain both biopsy and surgical samples from esophageal cancer patients and to compare gene expression profiles between these two kinds of samples. Here we report that artificially induced epithelial-mesenchymal transition (aiEMT) occurs in surgical samples. Its presence there has possibly interfered not only with microarray- or immunohistochemistory-based clinical research but also with basic research.

## Results

### Comparison of Expression Profiles between Biopsy and Surgically Resected Esophageal Tumor Samples Obtained from Different Cases

**Figure 1:**
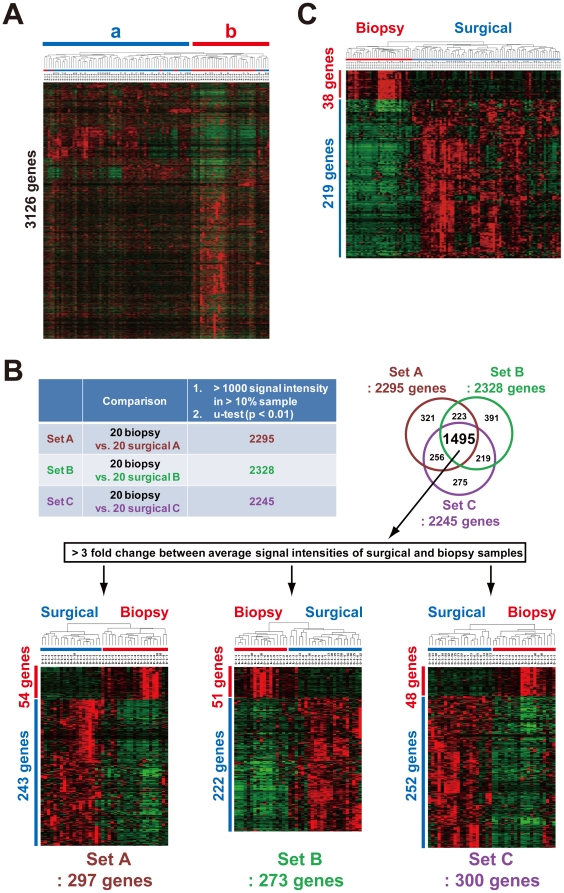
Comparison of expression profiles between biopsy and surgically resected esophageal tumor samples obtained from different cases. (A) Unsupervised clustering with 3,126 processed genes. Surgical (a) and biopsy sample clusters (b) are shown. (B) Comparison of expression profiles among three independent sets (A, B, and C): a randomly selected 20-biopsy sample set versus three surgical sample sets (A, B, and C) containing 20 independent cases. The number of differentially expressed genes selected by u-test (p<0.01): 2, 295 in set A, 2,328 in set B, and 2, 245 in set C (Upper). The number of differentially expressed genes with a 3-fold change between two average signal intensities: 297, 273, and 300. Clustering results with these gene sets (Lower). (C) Up-regulated genes in surgical or biopsy samples. By the use of all of the profiles under stringent selection conditions (see Materials and Methods), 38 and 219 genes were identified as up-regulated genes in the biopsy and surgical samples, respectively.

We first compared gene expression profiles between 35 fresh biopsy samples containing no necrotic lesion and 66 surgical esophageal tumor samples, which were obtained from a margin of the tumor after exposure for 4–7 hours under an ischemic condition, by unsupervised clustering with 3,126 processed genes (Materials and Methods). There was no significant difference in clinical or pathological stage distribution between these two sets of esophageal cancers because locally advanced tumors (Stage II or III) are major targets of both chemoradiotherapy and surgery [8]–[10]. Sixty of the 66 surgical samples (90.9%) and 29 of the 35 biopsy samples (82.9%) appeared in a (left) and b (right) sample cluster, respectively (Figure 1A). To investigate the number of differentially expressed genes between these two kinds of samples with reproducibility, we compared expression profiles among three independent sample sets (A, B, and C): another 20 biopsy sample set versus three surgical sample sets (A, B, and C) containing 20 randomly selected cases from the 66 cases (Figure 1B, upper). The number of differentially expressed genes selected by u-test (p<0.01) were 2, 295, 2,328, and 2, 245 in sets A, B, and C, respectively. Among these 3 sets, 1,495 genes (65.1% in A, 64.2% in B, and 66.6% in C) were commonly identified (Figure 1B, upper). Therefore, more than 20% (1,495/6,000, 24.9%) of the genes were differentially expressed between biopsy and surgical samples because the average number of detectable genes in each case was approximately 6,000. These results suggested that a large difference exists between the biopsy and surgical samples.

From the 1,495 genes, we further selected differentially expressed genes among the 3 sets that had a 3-fold change between two average signal intensities of each gene between the biopsy and surgical samples. From sets A, B, and C, 297, 273, and 300 genes were identified, respectively (Figure 1B, lower). More than 80% of these genes were over-expressed in the surgical samples, suggesting a preferential presence of artificial factors or a contamination of normal portions.

**Figure 2:**
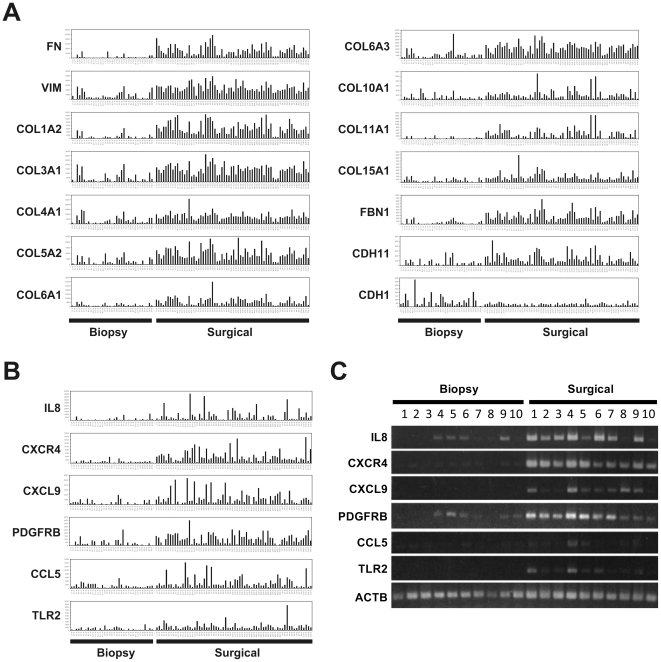
Representative EMT related genes over-expressed in surgically resected esophageal tumor samples. (A) Expression patterns of an epithelial cell marker E-cadherin (*CDH1*) and typical EMT markers including fibronectin (*FN*), vimentin (*VIM*), and collagens (*COLs*). (B) Expression patterns of 6 membrane signal transducers: a cytokine (*IL8*), two chemokines (*CXCL9* and *CCL5*), and three membrane type receptors (*CXCR4*, *PDGFRB*, and *TLR2*). (C) Semi-quantitative RT-PCR results of these 6 membrane transducers in representative samples.

To address the rationale for the difference, we finally selected genes that expressed preferentially in all the 35 biopsy or 66 surgical samples under stringent conditions with u-test (p<0.01), permutation test, and a 2-fold change, etc. (Materials and Methods). By this procedure, 38 and 219 genes were identified as up-regulated genes in the biopsy and surgical samples, respectively (Table S1 and Figure 1C). Interestingly, in the surgical samples, many EMT markers were found to be expressed preferentially and frequently. Microarray results of 13 representative EMT markers including fibronectin (FN), vimentin (VIM) and collagens (COLs) are shown in Figure 2A. Moreover, membrane signal transducers such as cytokine, chemokine, and receptors were also found to be up-regulated in the surgical samples. Representative microarray and RT-PCR results of *IL8*, *CXCR4*, *CXCL9*, *PDGFRB*, *CCL5*, and *TLR2*, respectively are shown in Figures 2B and 2C. In correspondence with EMT, E-cadherin (*CDH1*) was found to be down-regulated in the surgical samples (Figure 2A, right lowest).

### Comparison of Expression Profiles between Biopsy Samples and Surgical Resected Esophageal Tumor Samples Obtained from Identical Cases

**Figure 3:**
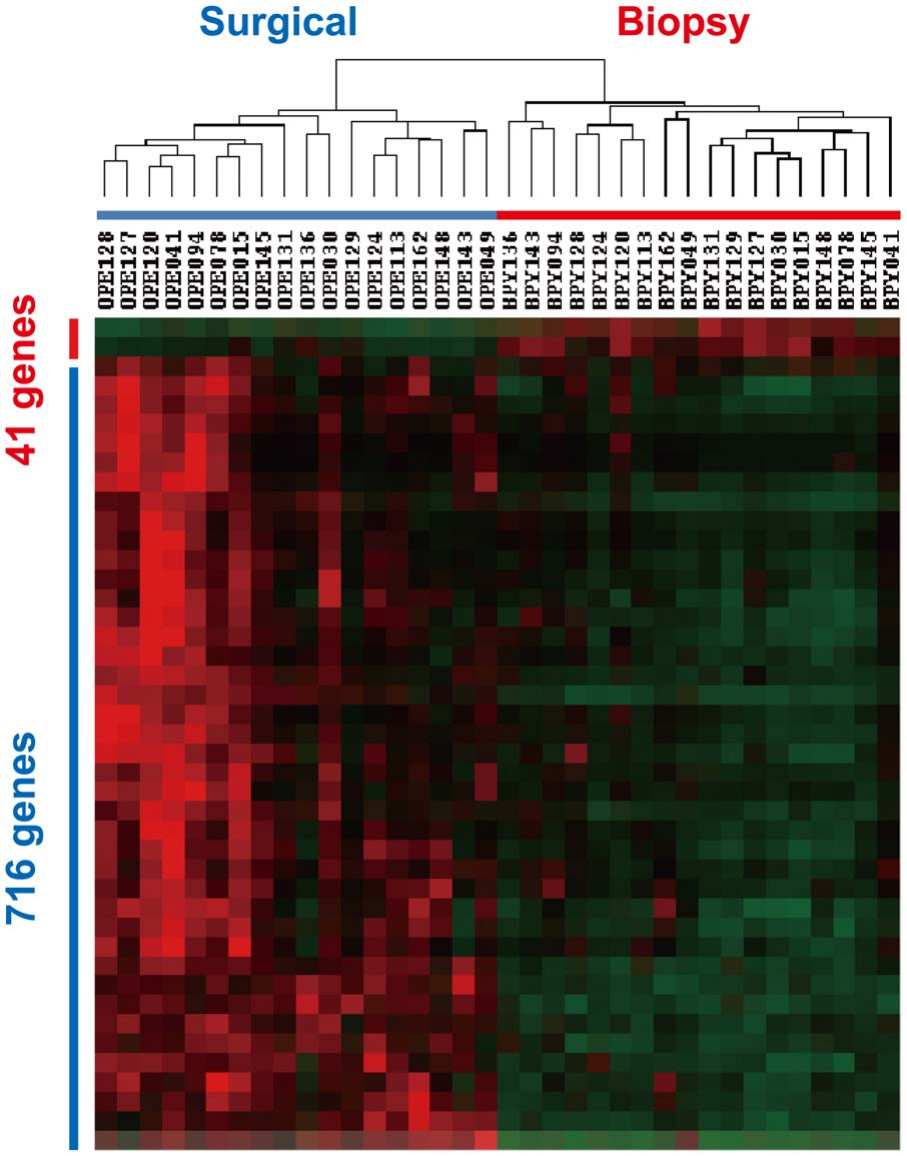
Up-regulated genes in biopsy and surgically resected esophageal tumor samples obtained from identical cases. By stringent selection (see Materials and Methods), 41 and 716 genes were identified as up-regulated genes in the biopsy and surgical samples, respectively.

**Figure 4:**
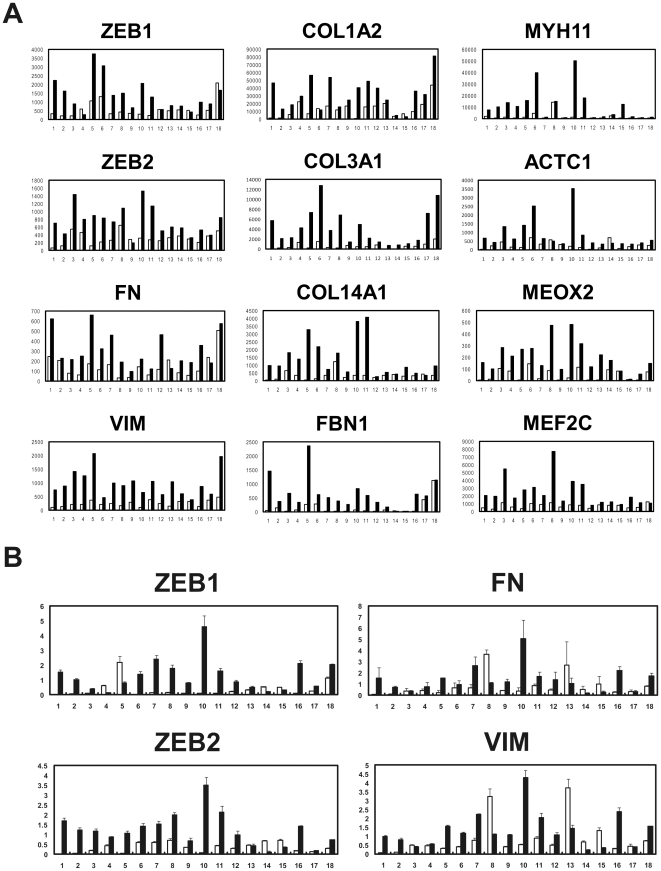
Representative EMT related genes also over-expressed in surgically resected esophageal tumor samples obtained from identical cases. (A) Expression patterns of 2 representative EMT regulators (*ZEB1* and *ZEB2*), 8 typical EMT markers including fibronectin (*FN*), vimentin (*VIM*), 3 collagens (*COL1A2*, *COL3A1*, and *COL14A1*), *FBN1*, *MYH11*, and *ACTC1*, and 2 EMT-related myogenic transcription factors (*MEOX2* and *MEF2C*). (B) Quantitative real-time RT-PCR results of *ZEB1, ZEB2, FN, and VIM*. Closed box: surgical sample; Open box: biopsy sample.

**Figure 5:**
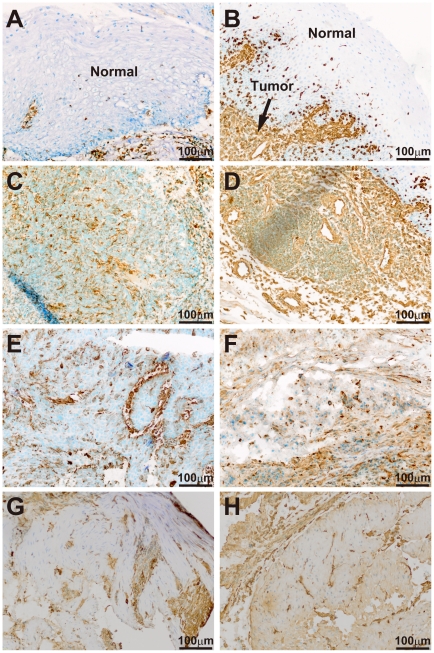
Immunohistochemistry (IHC) of a typical EMT marker vimentin in biopsy and surgically resected tissues. IHC of vimentin in an additional surgical sample, which contained normal portions, showed that normal esophageal epithelial cells were not stained, but invasive tumor cells were (A, B). In 3 out of 5 pairs of biopsy and surgical samples, over-expression of vimentin was observed in the surgical samples (biopsy: C, E, G; surgical: D, F, H).

In the same above way, we compared gene expression profiles between 18 biopsy and 18 surgically resected esophageal tumor samples, and selected 41 and 716 genes that were identified as up-regulated genes in the two kinds of samples, respectively (Table S2 and Figure 3). In accordance with the above results from different cases, many EMT markers and membrane signal transducers were also found to be up-regulated frequently in the surgical samples (Figure 4A). More importantly, two EMT regulators, *ZEB1* and *ZEB2*, and some EMT-related myogenic transcription factors including *MEOX2* and *MEF2C* were able to be selected as up-regulated genes in the surgical samples (Figures 4A). Quantitative real-time RT-PCR confirmed over-expression of *ZEB1*, *ZEB2, FN, and VIM* in the 18 surgical samples of identical cases (Figure 4B). The over-expression of *ZEB1* and *ZEB2* was also found in the 66 surgical samples of different cases (Figure S2), although these two EMT regulators could not be extracted from expression profiles under the above stringent conditions. SNAI1/Snail, SNAI2/Slug, ZEB1/ZFHX1A, ZEB2/SIP1/ZFHX1B, TWIST1/TWIST, and TWIST2 are representative EMT regulators [11], [ 12]. Among them, *TWIST1* as well as two *ZEBs* were over-expressed in the two sets of esophageal tumors (Figure S3). To investigate whether aiEMT in the mRNA levels affects immunohistochemistry (IHC), we performed IHC on a typical mesenchymal marker vimentin in biopsy and surgical samples of identical cases. First we determined conditions under which normal epithelial cell layers could not be stained, but tumor cells with EMT could be (Figures 5A, 5B), because undifferentiated layers (basal and parabasal) have been reported to express EMT-related genes including *VIM* [4]. In 3 out of 5 pairs of the samples examined, tumor lesions of a surgical sample were found to be stained more highly than those of a biopsy sample (Figures 5C–H); however, the remaining 2 pairs did not show such difference (data not shown). Therefore, the aiEMT that occurred in the surgical samples in the mRNA level was thought to affect only a subset of surgical samples in the level of EMT-related proteins.

### Over-expression of *ZEB1*, *ZEB2*, and *TWIST1* in Surgically Resected Normal Tissues

**Figure 6:**
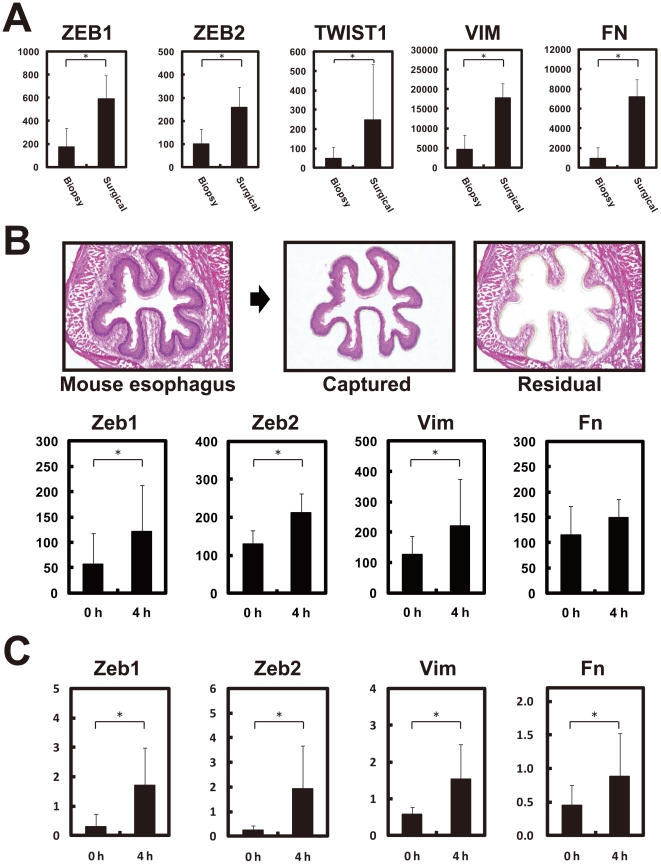
Over-expression of EMT regulators and markers in surgically resected normal tissues. (A) Over-expression of EMT-regulators (*ZEB1*, *ZEB2*, and *TWIST1*) and EMT-markers (*VIM* and *FN*) in surgically resected normal esophagus mucosa. (B) Induction of mouse *Zeb1*, *Zeb2*, *Vim*, and *Fn* under ischemic condition. After resection of mouse esophagus, we placed it on PBS for 0 or 4 hours at room temperature (under an ischemic condition), immediately made frozen sections, captured the epithelial cell layer (upper) by laser microdissection, amplified mRNA by TALPAT [24]–[28], and obtained expression profiles using Mouse Expression Array 430 2.0 (Affymetrix, Santa Clara, CA). Experiments were performed on 3 mice. The *Zeb1*, *Zeb2*, *Vim*, and *Fn* genes are induced 4 hours after resection (Lower). *P<0.05. (C) Quantitative real time RT-PCR of *Zeb1*, *Zeb2*, *Vim*, and *Fn*. Over-expression of *Zeb1*, *Zeb2*, *Vim*, and *Fn*, shown by microarray, was confirmed.

We obtained 4 biopsy samples and 5 surgical samples of non-cancerous tissues, and compared their expression profiles. In the same manner with the above expression profiles of tumor tissues (Figures 2, 4, S2, and S3), three EMT regulators (*ZEB1*, *ZEB2*, and *TWIST1*) and two typical EMT markers (*VIM* and *FN*) were found to be over-expressed in the 5 surgical samples (Figure 6A). Our previous report showing the involvement of ZEB2 and TWIST1 in the EMT of normal and malignant esophageal epithelial cells [9] supports the presence there of artificially induced EMT.

Finally, to investigate whether these 5 genes are induced in epithelial cells by surgical resection-related ischemia, we resected a mouse esophagus, and placed it on PBS for 0 or 4 hours, and immediately made frozen sections followed by laser-captured microdissection of the epithelial cell layers (Figure 6B, upper). Expression profiles of the mouse epithelial cell layers at 0 or 4 hours after resection revealed that mouse *Zeb1*, *Zeb2*, *Vim*, and *Fn* were induced 4 hours after resection (Figure 6B, lower). Quantitative real-time RT-PCR confirmed over-expression of *Zeb1*, *Zeb2*, *Vim*, and *Fn* after resection (Figure 6C). Since overall sensitivity of mouse affymetrix arrays is known to be lower than in humans, and the use of a small amount of RNA such as laser-captured subjects is also known to reduce microarray sensitivity, *Twist1* mRNA itself could not be detected in this mouse experiment (data not shown).

**Figure 7:**
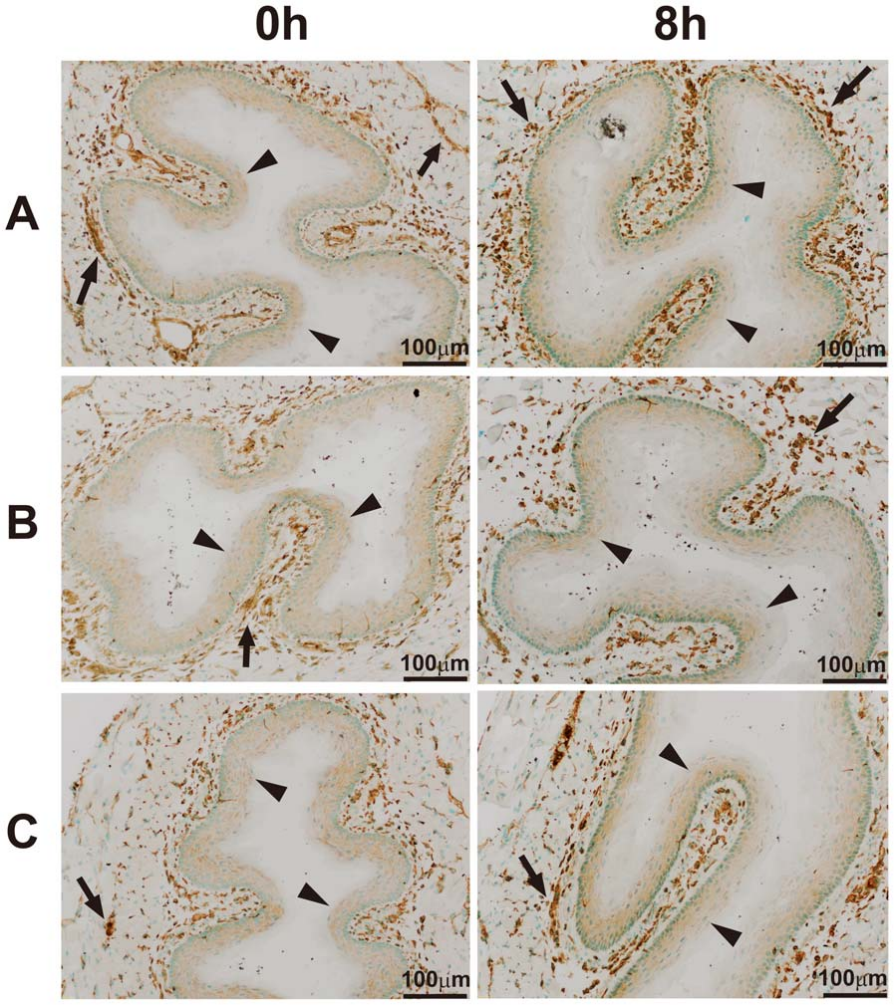
Immunohistochemistry (IHC) of a typical EMT marker vimentin in mouse esophagus. After resection of mouse esophagus, we placed it on PBS for 0, 4, 8 hours at room temperature (under an ischemic condition), immediately made frozen sections, and IHC of vimentin was performed under more sensitive conditions compared with Figure 5. Experiments were performed on 3 mice (A–C). Over-expression of vimentin in mouse esophageal epithelium was not observed even after 8 hours of exposure under an ischemic condition. Arrow: vimentin-positive smooth muscle, arrow head: mouse stratified esophageal epithelial cell layers.

To investigate whether aiEMT in the mRNA levels affects IHC, we performed IHC on a typical mesenchymal marker vimentin 8 hours after resection. Here we determined conditions under which normal epithelial cell layers are stained. In all of the 3 independent samples examined, normal epithelial cell layers were not found to be stained highly 8 hours after resection (Figures 7A–C). The discrepancy between the mRNA level and protein level can be explained by the two following reasons: 1) although undifferentiated layers (basal and parabasal) have been reported to express EMT-related genes including *VIM* [4], their expression levels were much lower than tumor (Figure 5B). 2) it may also be difficult to show the approximate 2-fold change in the mRNA level (Figures 6B, C) as in the protein level by IHC, because IHC is inferior to RT-PCR in both sensitivity and quantification.

All of the results suggest that EMT, especially in the mRNA level, is induced artificially in both normal and malignant epithelial cells by surgical resection-related events (ischemia-induced hypoxia and hyponutrition, and hypoxia-induced inflammation, etc.).

### Artificially Induced EMT (aiEMT) by Surgical Resection Prevents Microarray-based Subgroup Identification

**Figure 8:**
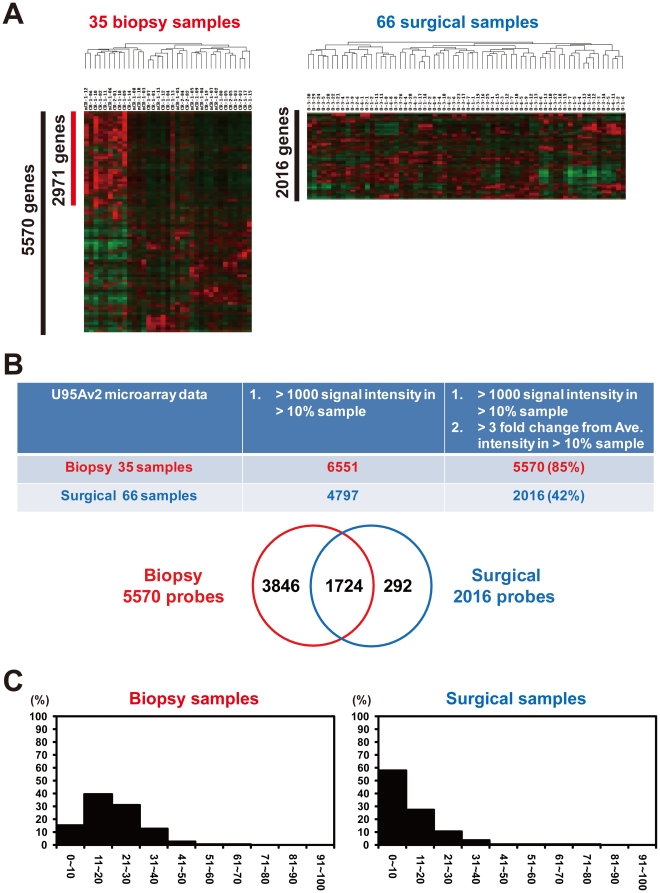
Artificially induced EMT prevents microarray-based subgroup identification. (A) Unsupervised clustering of 35 biopsy and 66 surgically resected esophageal tumor samples with 5,570 and 2,016 processed genes, respectively. A sample cluster with 2,971 genes appears only in the biopsy samples. (B) Comparison of the number of processed genes for unsupervised clustering between biopsy and surgical samples. The number of processed genes and commonly selected genes is indicated. (C) Frequency distribution for percentage of samples of finally processed-gene sets. Each distribution of 5, 570 genes in biopsy samples (Left) and 2,016 genes in surgical samples (Right) is indicated.

Identification of clinically significant subgroups is very important for personalized medicine and for drug development against intractable cases. When we used the expression profiles of the 35 biopsy samples obtained from patients treated by chemoradiotherapy [8], unsupervised clustering with 5,570 processed genes (Materials and Methods) identified a good responder group consisting of 9 patients (7/9, 78% showing complete response to chemoradiotherapy) from the 35 (Figure 8A, left). However, when the profiles of the 66 surgical samples were used, unsupervised clustering with 2,016 processed genes could not identify any subgroup (Figure 8A, right). Thus, biopsy sample expression profiles seemed to be more effective in subgroup identification than those of the surgical samples. In fact, we previously reported that biopsy sample expression profiles could distinguish long-term or short-term survivors by definitive chemoradiotherapy [8]; however, surgical sample expression profiles never identified poor prognostic subgroups with extensive lymph node metastasis [10]. Moreover, in the surgical samples, EMT was accelerated in 36 (85.7%) out of 42 esophageal cancers [9]. This high percentage seems to be caused by aiEMT.

To address the reason why subgrouping is difficult in surgical samples, we compared the number and distribution of each of the processed genes, which were used for unsupervised clustering. We first selected genes with a signal intensity of more than 1,000 in more than 10% of the samples. From 35 biopsy and 66 surgical samples, 6,551 and 4,797 genes, respectively, were selected. From these genes, we finally selected more than 3-fold changed genes by comparing the average signal intensity of each gene in more than 10% of the samples. In the 35 biopsy samples, 85% (5,570) of 6,551 first processed genes remained, whereas the number of final processed genes decreased from 4,797 first processed genes to 2,016 (42%) (Figure 8B, upper). Of the 2,016 finally processed genes in the surgical samples, 1,724 (86%) were included in the 5,570 finally processed genes in the biopsy samples; however, 3,846 (69%) of 5,570 genes were unique to the biopsy samples (Figure 8B, lower). Moreover, frequency distribution (for percentage of samples) of these two finally processed-gene sets shows that approximately 60% of the 2,016 processed genes in the surgical samples express in only a limited number of cases (0–10%) (Figure 8C). Accordingly, aiEMT in surgical samples may diminish the number of processed genes useful for subgroup identification.

## Discussion

We recently reported the presence of crosstalk between Hedgehog (Hh) and EMT signaling in normal and malignant epithelial cells of the esophagus [9]. In that report, *ZEB2* was shown to be a downstream gene of both a primary transcriptional transducer GLI1 in Hh signaling and of another EMT regulator, TWIST1, and that ZEB2 further up-regulated 5 chemokine or growth factor receptors, *PDGFRA*, *EDNRA*, *CXCR4*, *VEGFR2*, and *TRKB* (Figure S4). The Hh signal block inhibited esophageal keratinocyte differentiation and cancer cell invasion and growth. Accordingly, over-expression of *ZEB2* and *TWIST1* in surgical samples of both normal and tumor tissues can induce EMT, resulting in over-expression of representative EMT markers *VIM*, *FN*, and *COLs* (Figures 2, 4, 6, S2, and S3) and membrane signal transducers *IL8*, *CXCL4*, *CCL5*, *CXCR4*, *PDGFRB*, and *TLR2* (Figure 2). Over-expression of the membrane signal transducers can activate further down-stream cascades. This is a major reason for the large difference of expression profiles between biopsy and surgical samples (Figures 1 and 3).

Extensive contamination of normal mesenchymal portions in surgically resected tumor tissues can also explain the over-expression of those EMT regulators and EMT-related genes, even though trained pathologists carefully excised bulk tissue samples from the main tumor, leaving a clear margin from the surrounding normal tissue (Materials and Methods). However, the over-expression was also observed in surgically resected normal tissue and mouse epithelial cell layers 4 hours after resection (Figure 6). Therefore, we concluded that artificially induced EMT, termed aiEMT, occurred in both normal and malignant epithelial cells by the surgical resection-related events (ischemia-induced hypoxia, ischemia-induced hyponutrition, and hypoxia-induced inflammation, etc.) (Figure S1).

Recently, the hypoxia-inducible factors (HIF-1A or HIF-2A) have been reported to directly regulate TWIST1 [13], [ 14] and LOXL2, which reportedly stabilized an EMT regulator, SNAI1/SNAIL, through physical interaction on the SLUG domain and Snail's lysine residues K98 and K137 [15]. The SNAI1 binding site was also found in the 5’ promoter region of *ZEB2* [16]. Over-expression of both *HIF1A* and *LOXL2* was observed only in the surgically resected tumor tissues obtained from different cases (Figure S5). Moreover, other *HIF1* families (*HIF1B* and *HIF2A*) were never over-expressed in any of the surgical samples. Therefore, elucidation of the molecular mechanisms of aiEMT in surgical samples remains for future studies. However, we noted that ischemia-induced hypoxia and/or inflammation has been reported to release repression of NFκB [17], which regulates *ZEB1*, *ZEB2*, and *TWIST1* [18], [ 19] and that TGF-β signaling may be involved in aiEMT, because over-expression of *NFKB1* and *TGFBR2* was found in surgical samples (Figure S6).

As mentioned in the Introduction, surgical samples have been used as important subjects for clinical and basic cancer research for many years. Therefore, aiEMT in surgical samples may have possibly interfered with or prevented not only microarray- or immunohistochemistry-based clinical research (diagnostic marker identification, subgrouping, making predictors, and prognosis evaluation, etc.) but also basic research (making a signal pathway map, therapeutic target identification, etc.). This study will likely evoke fundamental misinterpretation including underestimation of the prognostic evaluation power of markers by overestimation of EMT in past cancer research, and will provide some advice for the near future as follows: 1) Understanding how long the tissues were under an ischemic condition (from start of resection to stock or RNA preparation). The total amount of time should never exceed 4 hours. 2) Prevalence of biopsy samples for *in vivo* expression profiling with low biases on basic and clinical research; for example, for clinical outcome prediction of not only neoadjuvant but also adjuvant chemotherapy, radiotherapy, and chemoradiotherapy such as in previous reports [8], [ 20]–[23]. 3) Checking cancer cell contents and normal- or necrotic-tissue contamination in biopsy samples for the prevalence. In sampling by a needle biopsy, tumor portions (2mm X 2mm) should be obtained from a margin (periphery) of the tumor by exclusion of central necrotic lesions under endoscopy. If necrotic lesions were severely contaminated in the samples, those samples should be excluded by quantifying and qualifying RNA. If the samples contained extensive normal lesions, such samples can be excluded by the expression profile-based scoring method using normal and/or tumor specific genes.

## Materials and Methods

### Tissue Samples

All esophageal cancer (squamous cell carcinomas) and non-cancerous tissues were provided by the Central Hospital or East Hospital at the National Cancer Center after obtaining written informed consent from each patient and approval by the Center's Ethics Committee.

All surgical samples were obtained from patients without neoadjuvant therapy, and all biopsy samples were obtained before treatment. For the surgical samples, trained pathologists carefully excised bulk tissue samples from the main tumor, leaving a clear margin from the surrounding normal tissue. Thus, we obtained surgical samples from a margin (periphery) of the tumor. For the needle biopsy samples, tumor portions (2 mm X 2 mm) were obtained under endoscopy from a margin of the tumor by exclusion of any central necrotic lesions. If the samples were severely contaminated by necrotic lesions, those samples were excluded by quantifying and qualifying RNA. If the samples contained extensive normal lesions, we excluded such samples by the expression profile-based scoring method using normal and/or tumor specific genes (in preparation).

The overall process of an esophageal cancer operation requires much time. Therefore, surgical samples were excised from a margin of the tumor by trained pathologists after exposure for 4–7 hours under an ischemic condition, and were immediately frozen at −80°C until use. On the contrary, needle biopsy samples resected under endoscopy were immediately frozen at −80°C until use. Clinicopathological information is given in Tables S3, S4, S5.

### Laser Microdissection followed by RNA Extraction and Amplification

Cryostat sections (8µm) of frozen mouse esophageal samples were laser-microdissected with the mmi CellCut system (MMI Inc., Rockledge, FL). Total RNA was isolated by suspending the cells in an ISOGEN lysis buffer (Nippon Gene, Toyama, Japan) followed by precipitation with isopropanol. RNA was amplified by an efficient method of high-fidelity mRNA amplification, called TALPAT (T7 RNA polymerase promoter-attached, adaptor ligation-mediated, and PCR amplification followed by *in vitro* T7-transcription) [24–28].

### Microarray Analysis

Gene expression profiles were obtained from 166 samples: tumor sets (different cases) of independent 35 and 20 biopsy samples and 66 surgical samples, another tumor set (identical case) of 18 biopsy samples and 18 surgical samples, a normal set of 4 biopsy samples and 5 surgical samples. Total RNAs extracted from the bulk tissue samples were biotin-labeled and hybridized to high-density oligonucleotide microarrays (Human Genome U95Av2 or U133PLUS2.0 Array, Affymetrix, Santa Clara, CA, USA) in accordance with the manufacturer's instructions. For laser-captured mouse esophageal epithelial cell layers, Mouse Genome 430 2.0 Array was used. The scanned data of the arrays were processed by Affymetrix Microarray Suite version 4.0 or 5.0, which scaled the average intensity of all the genes on each array to a target signal of 1,000 to reliably compare variable multiple arrays. All the microarray data have been deposited in a MIAME compliant database, GEO; the accession number SuperSeries GSE22954.

### Gene Selection from Microarray Data and Hierarchical Clustering

Hierarchical clustering is widely used as one of the unsupervised learning methods. Hierarchical clustering of microarray data was performed by the use of GeneSpring (Agilent Technologies Ltd., CA, USA), Microsoft EXCEL, and Cluster & TreeView software [29].For unsupervised clustering (Figures 1A and 8A), we first selected genes with a signal intensity of more than 1,000 in more than 10% of the samples, and from these genes, we finally selected more than 3-fold changed genes by comparing the average signal intensity of each gene in more than 10% of the samples. For overexpressed genes in the surgical or biopsy samples, we first selected genes by u-test (p<0.01), permutation test, and 2- or 3-fold change between the average signal intensities of the two sets of samples, and from the first selected genes we finally selected genes with more than 1,000 in average signal intensity.

### Semi-quantitative and Quantitative RT-PCR

Total RNA was isolated by suspending the cells in Isogen lysis buffer (Nippon Gene, Toyama, Japan) followed by precipitation with isopropanol. RT-PCR was carried out using primer sets designed for detecting the 3′ side of cDNA of each human gene: for *IL8*, 5′- TGCCAAGGAGTGCTAAAG -3′ and 5′- CTCCACAACCCTCTGCAC-3′, for *CXCR4*, 5′-TGTATGTCTCGTGGTAGGAC-3′ and 5′-AGACTGTACACTGTAGGTGC-3′, for CXCL9, 5′-ACAAAGAAAATATTTCAAATTACAAGG-3′ and 5′-GGGAACGGTGAAGTACTAAGC-3′, for *PDGFRB*, 5′-ACTGCCCAGACCTAGCAGTG-3′ and 5′-CAGGGAAGTAAGGTGCCAAC-3′, for CCL5, 5′-CCCCGTGCCCACATCAAGGAGTATTT-3′ and 5′-CGTCCAGCCTGGGGAAGGTTTTTGTA-3′, for *TLR2*, 5′-CCAGCAGGAACATCTGCTAT-3′ and 5′-TCCAGGTAGGTCTTGGTGTT-3′, for *ZEB1*, 5′-CGTCTCTTTCAGCATCACCA-3′ and 5′-ATGGGAGACACCAAACCAAC-3′, for *ZEB2*, 5′-CATGACGTTGATCATTTGGGC-3′ and 5′-CGAGCATGGTCATTTTCAAAAG-3′, for *FN,* 5′-CGGGGGAAATAATTCCTGTG-3′ and 5′-CCTTGCAGGCAATCTCTTTG-3′, for *VIM,* 5′-GCTTTCAAGTGCCTTTCTGC-3′ and 5′-GTTGGTTGGATACTTGCTGG-3′, and for *ACTB* (β-actin), 5′-TCATCACCATTGGCAATGAG-3′ and 5′-CACTGTGTTGGCGTACAGGT-3′. Primer sets for detecting each mouse gene were also designed: for *Zeb1,* 5′-TAACATTTATACTTGCCTCC-3′ and 5′-GCTAAGGGAATGAGTTATGG-3′, for *Zeb2,* 5′-ACCAAATCAGACCACGAGGA-3′ and 5′-GCCCCTTCTGTCCCTCTCTA-3′, for *Fn,* 5′-CCGTGGGATGTTTTGAGACT-3′ and 5′-GGCAAAAGAAAGCAGAGGTG-3′, for *Vim*, 5′-ACGGTTGAGACCAGAGATGG-3′ and 5′-CGTCTTTTGGGGTGTCAGTT-3′ , and for *ActB,* 5′-GCTCTTTTCCAGCCTTCCTT-3′ and 5′-GTACTTGCGCTCAGGAGGAG-3′. For semi-quantitative RT-PCR, we showed data within linear range by performing 25–35 cycles of PCR. Quantitative real-time PCR was performed by a Bio-Rad iCycler with iQ Syber Green Supermix (Bio-Rad, Hercules, CA, USA) as directed by the manufacturer. The value of 1/2N (N: the number of PCR cycles corresponding to the onset of the linear amplification of each gene product) was calculated as a relative mRNA expression level of each gene normalized to *ACTB*. The data from 2 independent analyses for each sample were averaged.

### Immunohistochemistry

For immunohistochemical staining of frozen sections of human and murine esophagus, specimens that were embedded in a TissueTek OCT medium (VWR Scientific Products, West Chester, CA) and stocked at −80°C until use were cut into 8µm sections, which were then left for 30 min at room temperature followed by fixing in 4% paraformaldehyde for 20 min at room temperature. Endogenous peroxidase activity was inhibited with 3% H_2_O_2_ in methanol for 30 min. Blocking was carried out with Vectastain ABC Elite Kit (Vector Laboratories, Burlingame, CA) for 30 min at room temperature. Sections were incubated for 60 min at room temperature with diluted mouse monoclonal antibody directed against human vimentin (N1521, DAKO, Carpinteria, CA) or rabbit polyclonal antibody directed against mouse vimentin (#3932, Cell Signaling Technology Japan, Tokyo, Japan). After washing sections with PBS, biotinylated secondary antibodies were applied for 30 min at room temperature. Detection was carried out by using Vectastain ABC Elite Kit (Vector Laboratories) and the DAB system (DAKO, Tokyo), and the sections were counter-stained with 1% Methyl Green. (Sigma, Saint Luis, MO)

## Supporting Information

Figure S1 Schema of artificial factors during surgical resection and sample transportation. Biopsy samples are small, much fresher, with low contamination of normal portions compared to surgical samples, whereas some artificial factors such as ischemia, hypoxia, hyponutrition, and cold stress possibly occur during surgical resection and sample transportation.
(TIF)

Figure S2 Expression levels of *ZEB1* and *ZEB2* in two sets of biopsy and surgical samples (different and identical cases). Over-expression of both genes is observed in surgically resected esophageal tumors, except *ZEB2* in the different cases. *P<0.05.
(TIF)

Figure S3 Expression levels of *TWIST1* in two sets of biopsy and surgical samples (different and identical cases). Over-expression of *TWIST1* is observed in surgically resected esophageal tumors. *P<0.05.
(TIF)

Figure S4 Schema of crosstalk between Hh and EMT signal pathways in esophageal cancers. The primary transcriptional factor GLI1 and an EMT regulator TWIST1 regulate another EMT regulator ZEB2, which activates any gene including membrane type receptors (*PDGFRA*, *EDNRA*, *CXCR4*, *VEGFR2*, and *TRKB*) [9].
(TIF)

Figure S5 Expression levels of *HIF1A*, *HIF1B*, *HIF2A*, and *LOXL2* in two sets of biopsy and surgical samples (different and identical cases). Over-expression of *HIF1A* and its target *LOXL2* is observed only in surgically resected esophageal tumors (different cases).
(TIF)

Figure S6 Expression levels of *NFKB1* and *TGFBR2* in two sets of biopsy and surgically resected tumor samples (different and identical cases) and in biopsy and surgically resected non-cancerous tissues (normal). Over-expression of *NFKB1* and *TGFBR2* is observed in all the sets of surgically resected samples. *P<0.05.
(TIF)

Table S1 219 up-regulated genes in 66 surgically resected esophageal tumors.
(DOC)

Table S2 716 up-regulated genes in 18 surgically resected esophageal tumors.
(DOC)

Table S3 Clinicopathological information of biopsy samples from different cases with esophageal squamous cell carcinoma.
(DOC)

Table S4 Clinicopathological information of surgical samples from different cases with esophageal squamous cell carcinoma.
(DOC)

Table S5 Clinicopathological information of biopsy and surgical samples from different cases with esophageal squamous cell carcinoma.
(DOC)
