# Supplementary figures and images for: Artificially Induced Epithelial-Mesenchymal Transition in Surgical Subjects: Its Implications in Clinical and Basic Cancer Research
Source: PLoS One. 2011 Apr 21;6(4):e18196. doi: 10.1371/journal.pone.0018196 (PMC3080870; doi:10.1371/journal.pone.0018196)

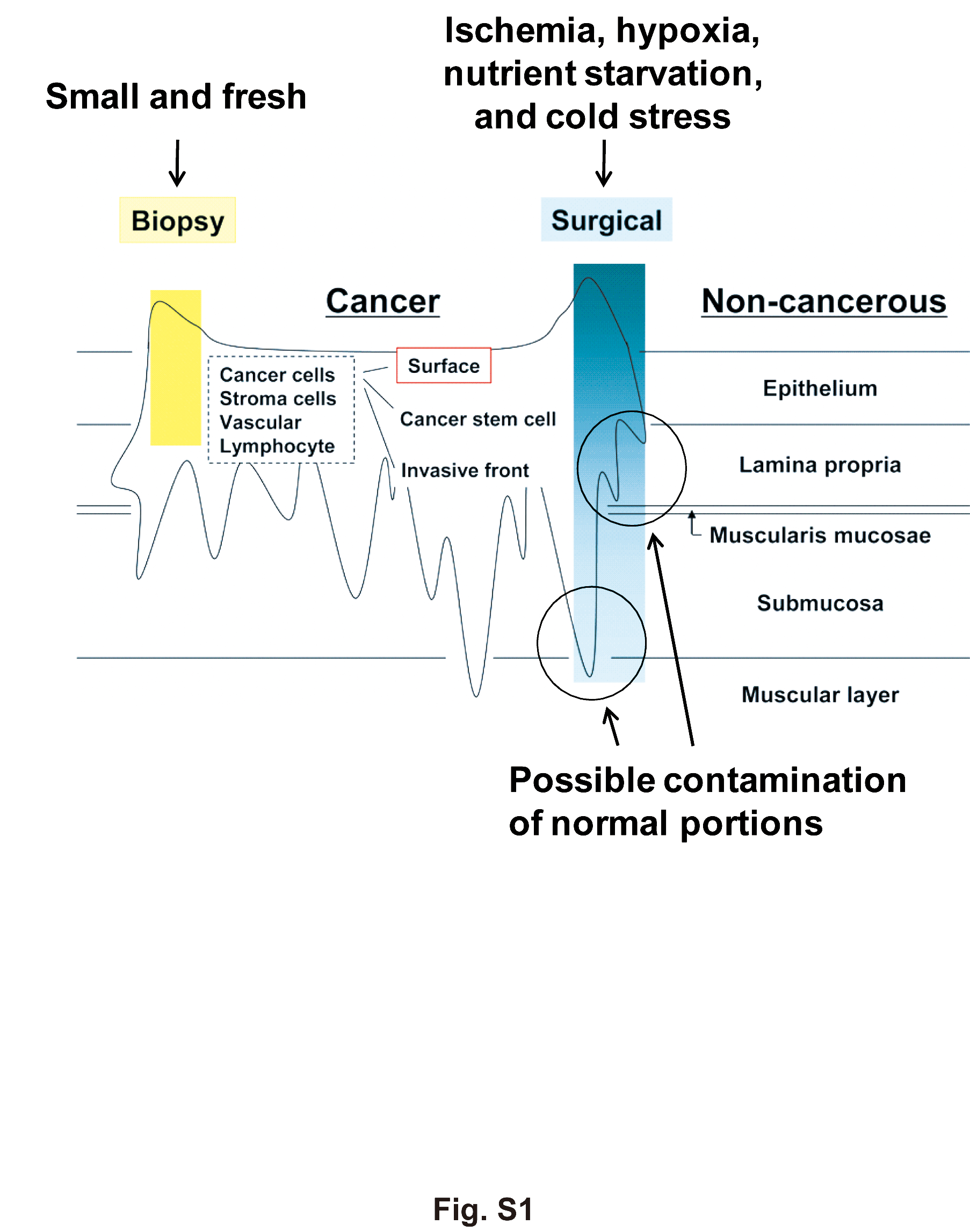

Supplement: Figure S1 — Schema of artificial factors during surgical resection and sample transportation. Biopsy samples are small, much fresher, with low contamination of normal portions compared to surgical samples, whereas some artificial factors such as ischemia, hypoxia, hyponutrition, and cold stress possibly occur during surgical resection and sample transportation. (TIF) [file pone.0018196.s001.tif]

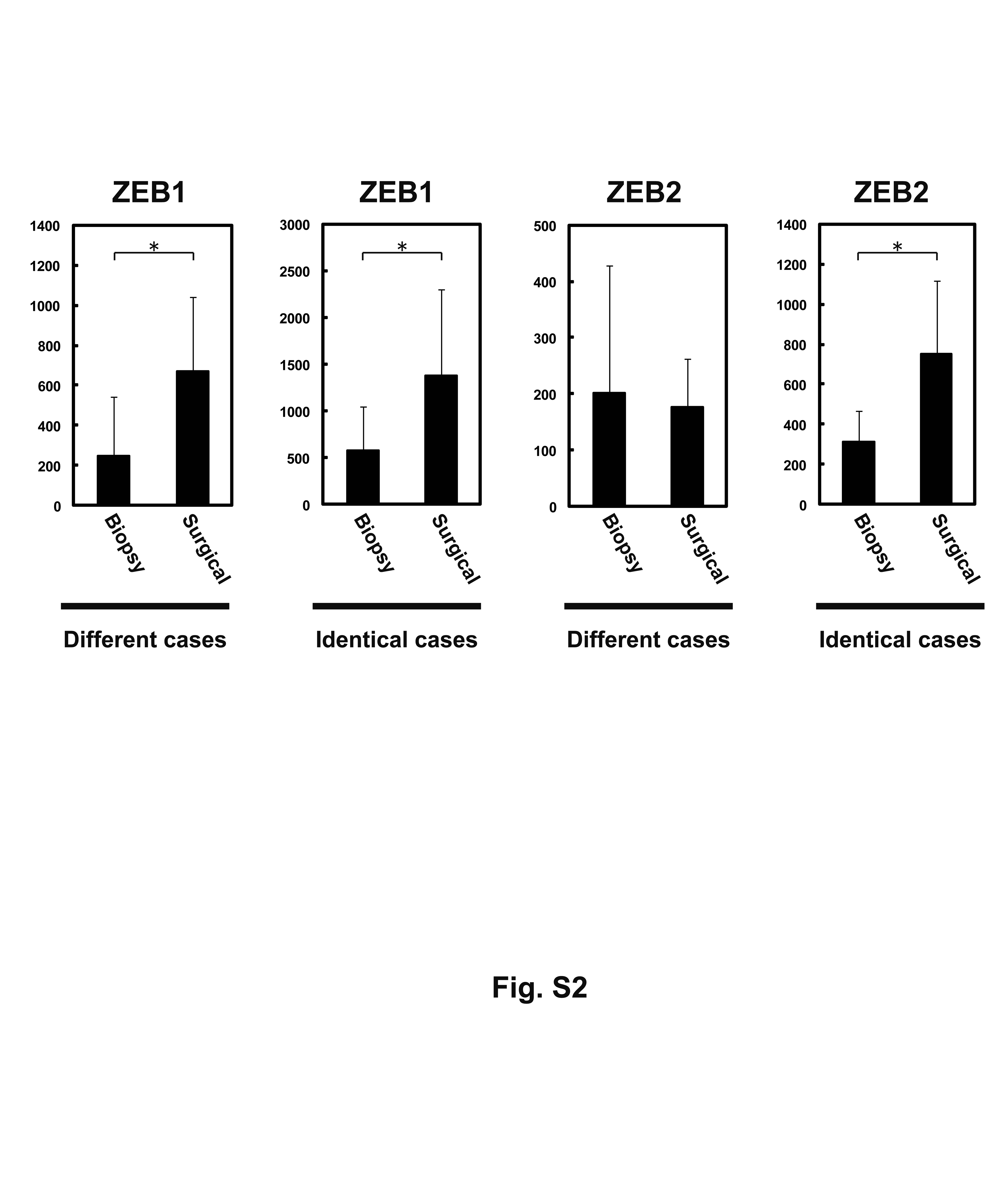

Supplement: Figure S2 — Expression levels of ZEB1 and ZEB2 in two sets of biopsy and surgical samples (different and identical cases). Over-expression of both genes is observed in surgically resected esophageal tumors, except ZEB2 in the different cases. *P<0.05. (TIF) [file pone.0018196.s002.tif]

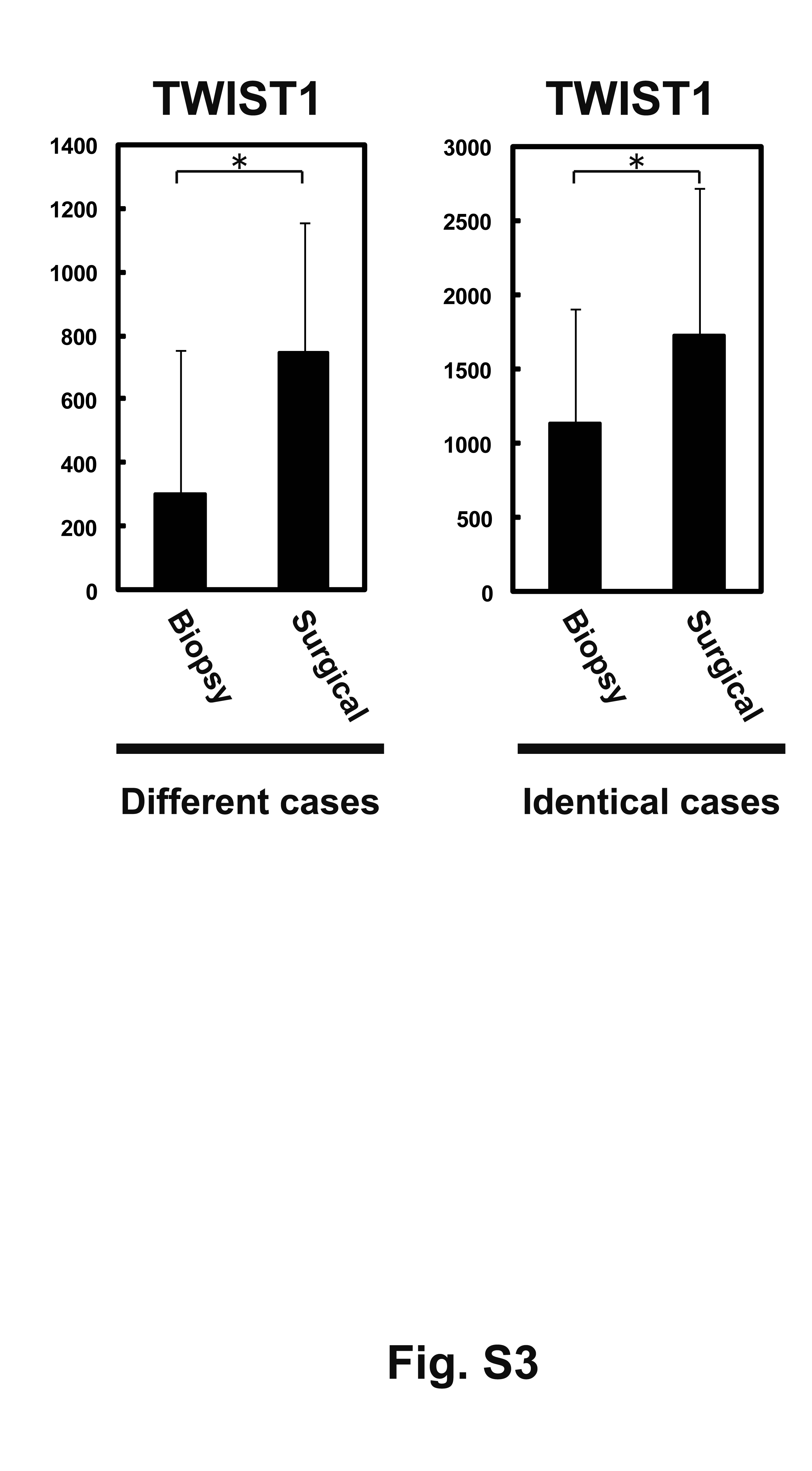

Supplement: Figure S3 — Expression levels of TWIST1 in two sets of biopsy and surgical samples (different and identical cases). Over-expression of TWIST1 is observed in surgically resected esophageal tumors. *P<0.05. (TIF) [file pone.0018196.s003.tif]

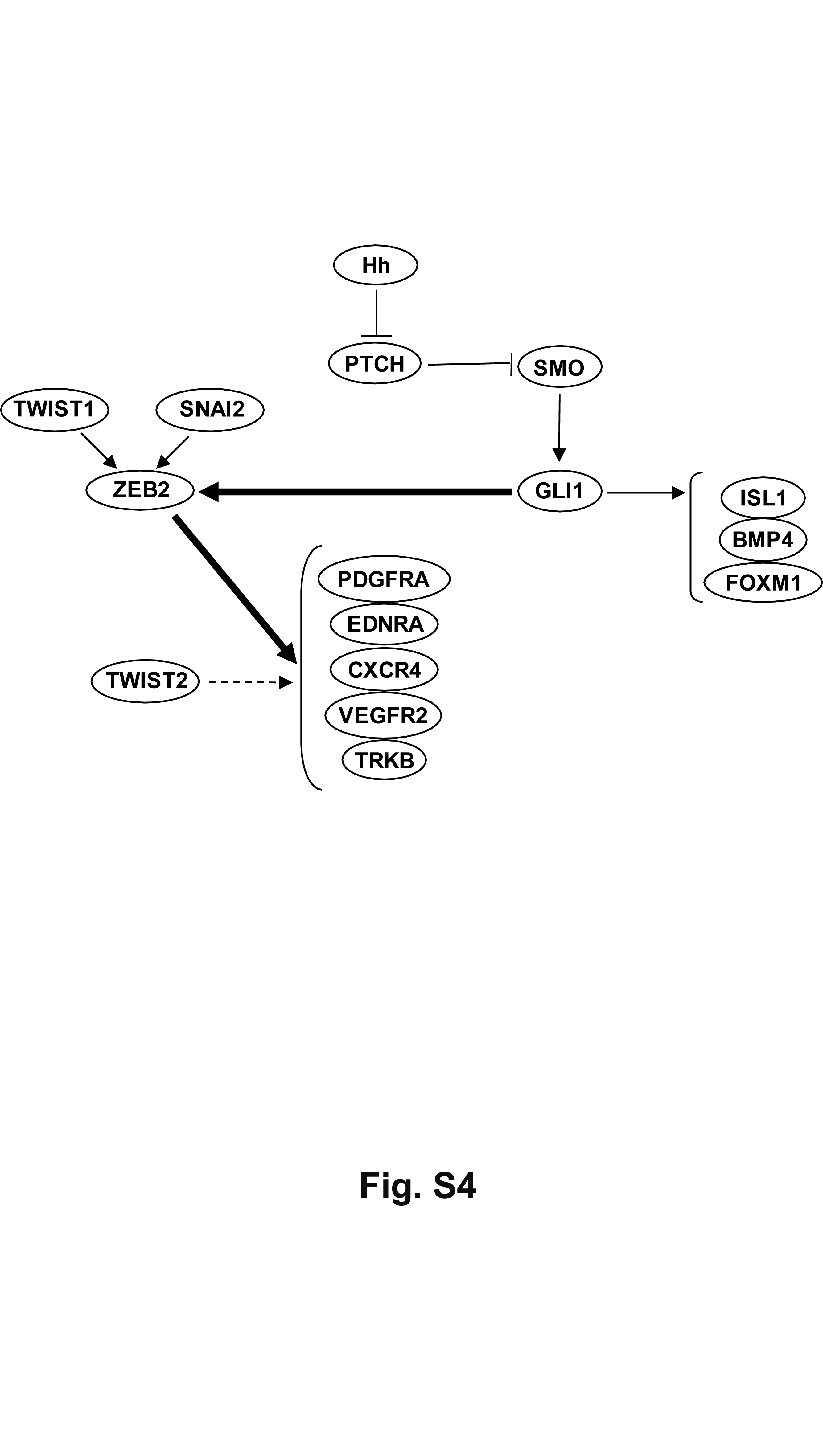

Supplement: Figure S4 — Schema of crosstalk between Hh and EMT signal pathways in esophageal cancers. The primary transcriptional factor GLI1 and an EMT regulator TWIST1 regulate another EMT regulator ZEB2, which activates any gene including membrane type receptors (PDGFRA, EDNRA, CXCR4, VEGFR2, and TRKB) [9]. (TIF) [file pone.0018196.s004.tif]

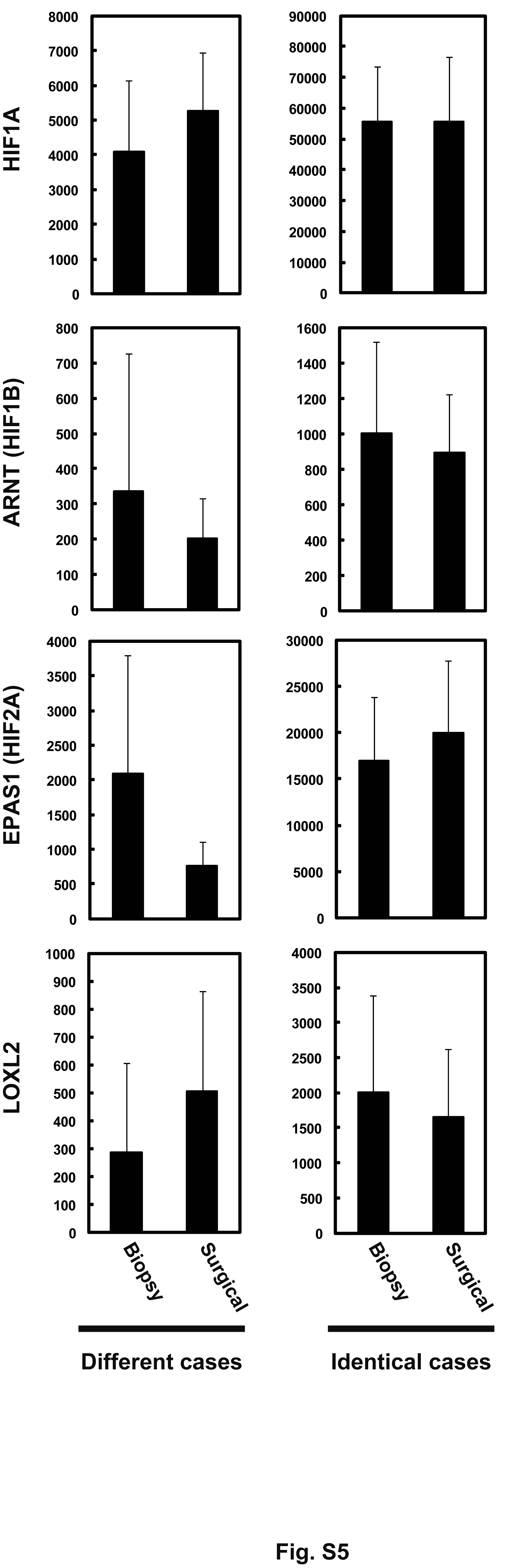

Supplement: Figure S5 — Expression levels of HIF1A, HIF1B, HIF2A, and LOXL2 in two sets of biopsy and surgical samples (different and identical cases). Over-expression of HIF1A and its target LOXL2 is observed only in surgically resected esophageal tumors (different cases). (TIF) [file pone.0018196.s005.tif]

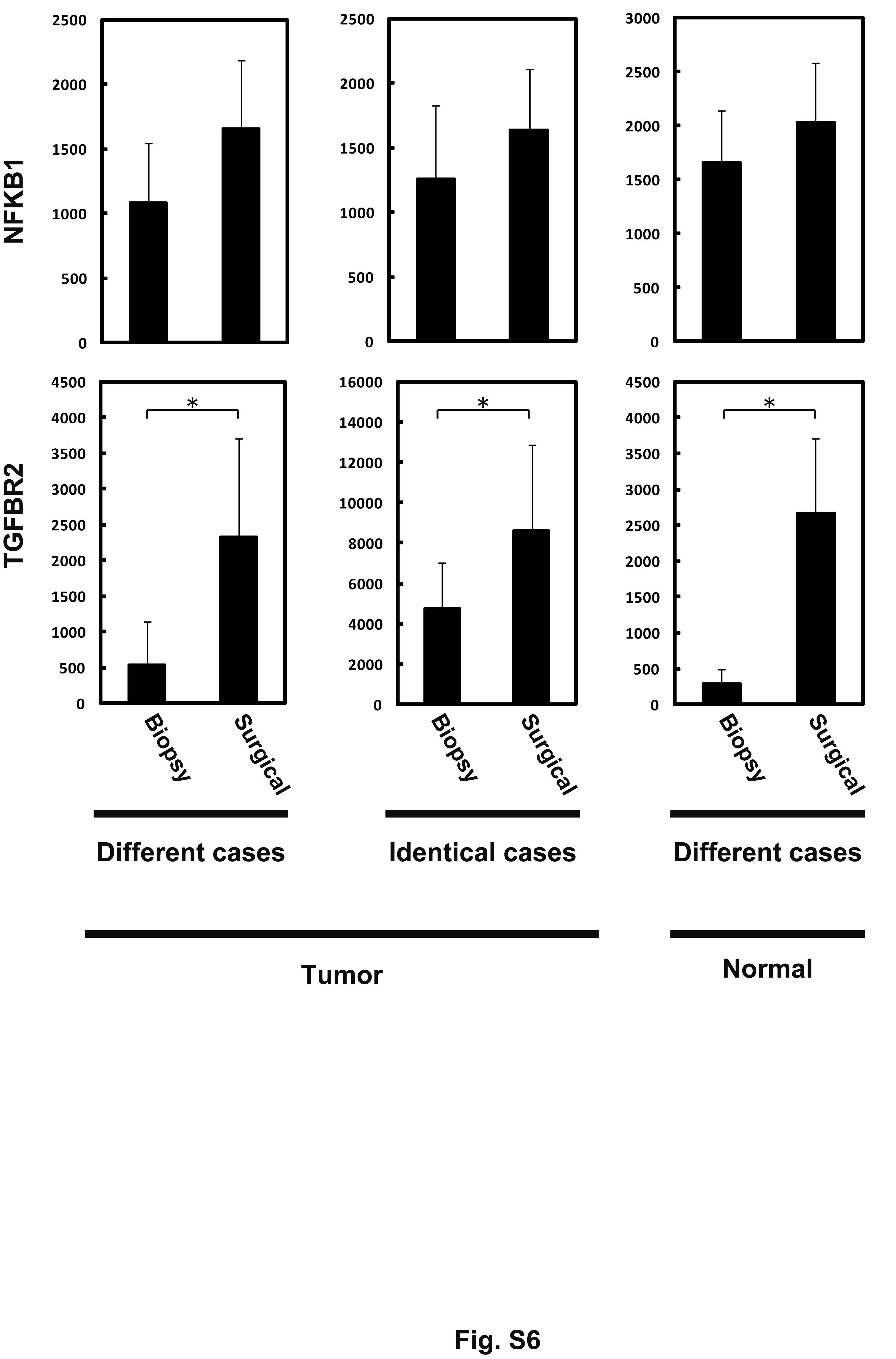

Supplement: Figure S6 — Expression levels of NFKB1 and TGFBR2 in two sets of biopsy and surgically resected tumor samples (different and identical cases) and in biopsy and surgically resected non-cancerous tissues (normal). Over-expression of NFKB1 and TGFBR2 is observed in all the sets of surgically resected samples. *P<0.05. (TIF) [file pone.0018196.s006.tif]
