# Supplementary material for: Artificially Induced Epithelial-Mesenchymal Transition in Surgical Subjects: Its Implications in Clinical and Basic Cancer Research
Source: PLoS One. 2011 Apr 21;6(4):e18196. doi: 10.1371/journal.pone.0018196 (PMC3080870; doi:10.1371/journal.pone.0018196)
Supplement: Table S1 — 219 up-regulated genes in 66 surgically resected esophageal tumors. (DOC) [file pone.0018196.s007.doc]

Table S1. 219 up-regulated genes in 66 surgically resected esophageal tumors

| Probe set ID | Gene symbol | Entrez gene ID | Average signal intensity | | Ratio (Surgical/Biopsy) |
| --- | --- | --- | --- | --- | --- |
|  |  |  | Biopsy | Surgical |  |
| 1197_at | ACTG2 | 72 | 199.48 | 4100.77 | 20.56 |
| 32313_at | TPM2 | 7169 | 655.93 | 8011.41 | 12.21 |
| 608_at | APOE | 348 | 893.55 | 7899.11 | 8.84 |
| 37892_at | COL11A1 | 1301 | 414.32 | 3548.09 | 8.56 |
| 311_s_at | FN1 | 2335 | 1932.48 | 16138.22 | 8.35 |
| 31720_s_at | FN1 | 2335 | 2106.51 | 15873.33 | 7.54 |
| 39145_at | MYL9 | 10398 | 611.26 | 4335.16 | 7.09 |
| 37279_at | GEM | 2669 | 337.26 | 2255.52 | 6.69 |
| 37402_at | RNASE1 | 6035 | 276.32 | 1830.88 | 6.63 |
| 41764_at | APOC1 | 341 | 618.74 | 4011.49 | 6.48 |
| 767_at | MYH11 | 4629 | 1924.80 | 11786.42 | 6.12 |
| 32305_at | COL1A2 | 1278 | 1056.13 | 6453.28 | 6.11 |
| 32847_at | MYLK | 4638 | 471.46 | 2800.63 | 5.94 |
| 32314_g_at | TPM2 | 7169 | 762.55 | 4491.92 | 5.89 |
| 38796_at | C1QB | 713 | 852.29 | 4856.28 | 5.70 |
| 39822_s_at | GADD45B | 4616 | 298.00 | 1681.53 | 5.64 |
| 38052_at | F13A1 | 2162 | 232.11 | 1290.16 | 5.56 |
| 36575_at | RGS1 | 5996 | 722.72 | 3955.43 | 5.47 |
| 39690_at | PDLIM3 | 27295 | 256.90 | 1404.84 | 5.47 |
| 31719_at | FN1 | 2335 | 4042.81 | 21634.72 | 5.35 |
| 1369_s_at | IL8 | 3576 | 1448.83 | 7658.91 | 5.29 |
| 1405_i_at | CCL5 | 6352 | 287.10 | 1513.77 | 5.27 |
| 649_s_at | CXCR4 | 7852 | 783.35 | 4081.06 | 5.21 |
| 31438_s_at | CD163 | 9332 | 336.44 | 1722.31 | 5.12 |
| 41123_s_at | ENPP2 | 5168 | 336.30 | 1707.56 | 5.08 |
| 35965_at | HSPA6 | 3310 | 295.02 | 1495.94 | 5.07 |
| 32535_at | FBN1 | 2200 | 544.18 | 2754.00 | 5.06 |
| 36629_at | TSC22D3 | 1831 | 1025.82 | 5155.76 | 5.03 |
| 37200_at | FCGR3A | 2214 | 505.87 | 2534.15 | 5.01 |
| 38111_at | VCAN | 1462 | 698.44 | 3416.04 | 4.89 |
| 37407_s_at | MYH11 | 4629 | 3650.74 | 17827.46 | 4.88 |
| 36627_at | SPARCL1 | 8404 | 2214.79 | 10413.02 | 4.70 |
| 36889_at | FCER1G | 2207 | 349.40 | 1537.41 | 4.40 |
| 41779_at | RGS16 | 6004 | 361.80 | 1586.70 | 4.39 |
| 1451_s_at | POSTN | 10631 | 1843.57 | 8073.65 | 4.38 |
| 1814_at | TGFBR2 | 7048 | 538.72 | 2332.43 | 4.33 |
| 39114_at | C10orf10 | 11067 | 588.41 | 2540.03 | 4.32 |
| 40520_g_at | PTPRC | 5788 | 328.87 | 1415.33 | 4.30 |
| 38772_at | CYR61 | 3491 | 616.62 | 2637.90 | 4.28 |
| 32307_s_at | COL1A2 | 1278 | 4021.99 | 17205.02 | 4.28 |
| 37099_at | ALOX5AP | 241 | 236.89 | 1008.45 | 4.26 |
| 129_g_at | CTSK | 1513 | 268.35 | 1138.76 | 4.24 |
| 38112_g_at | VCAN | 1462 | 580.22 | 2444.25 | 4.21 |
| 35832_at | SULF1 | 23213 | 698.11 | 2920.37 | 4.18 |
| 658_at | THBS2 | 7058 | 462.42 | 1898.01 | 4.10 |
| 40518_at | PTPRC | 5788 | 766.13 | 3123.67 | 4.08 |
| 671_at | SPARC | 6678 | 6594.17 | 26585.04 | 4.03 |
| 32542_at | FHL1 | 2273 | 677.02 | 2728.91 | 4.03 |
| 33924_at | DENND5A | 23258 | 339.43 | 1364.67 | 4.02 |
| 38326_at | G0S2 | 50486 | 428.44 | 1710.76 | 3.99 |
| 36931_at | TAGLN | 6876 | 3680.69 | 14623.40 | 3.97 |
| 41739_s_at | CALD1 | 800 | 286.78 | 1138.08 | 3.97 |
| 37032_at | NNMT | 4837 | 1581.66 | 6273.11 | 3.97 |
| 38466_at | CTSK | 1513 | 958.71 | 3761.45 | 3.92 |
| 128_at | CTSK | 1513 | 422.48 | 1616.21 | 3.83 |
| 38722_at | COL6A1 | 1291 | 1695.36 | 6423.65 | 3.79 |
| 36993_at | PDGFRB | 5159 | 700.31 | 2630.93 | 3.76 |
| 36791_g_at | TPM1 | 7168 | 1814.46 | 6809.40 | 3.75 |
| 661_at | GAS1 | 2619 | 558.72 | 2087.37 | 3.74 |
| 38650_at | IGFBP5 | 3488 | 2043.92 | 7634.60 | 3.74 |
| 37219_at | CXCL9 | 4283 | 367.11 | 1370.65 | 3.73 |
| 39069_at | AEBP1 | 165 | 1037.90 | 3795.65 | 3.66 |
| 39760_at | QKI | 9444 | 307.70 | 1124.29 | 3.65 |
| 35926_s_at | LYZ | 4069 | 2642.25 | 9491.57 | 3.59 |
| 1693_s_at | TIMP1 | 7076 | 4782.67 | 17092.53 | 3.57 |
| 659_g_at | THBS2 | 7058 | 842.23 | 3009.55 | 3.57 |
| 40202_at | KLF9 | 687 | 1194.99 | 4252.72 | 3.56 |
| 1396_at | IGFBP5 | 3488 | 1436.34 | 5092.08 | 3.55 |
| 32227_at | SRGN | 5552 | 563.54 | 1997.62 | 3.54 |
| 32783_at | FBLN2 | 2199 | 358.22 | 1260.45 | 3.52 |
| 1005_at | DUSP1 | 1843 | 752.95 | 2566.68 | 3.41 |
| 38420_at | COL5A2 | 1290 | 2911.25 | 9651.75 | 3.32 |
| 32773_at | HLA-DQA1 | 3117 | 1403.07 | 4591.17 | 3.27 |
| 39593_at | FGL2 | 10875 | 381.94 | 1247.03 | 3.26 |
| 37023_at | LCP1 | 3936 | 515.03 | 1671.20 | 3.24 |
| 38363_at | TYROBP | 7305 | 1856.74 | 5951.43 | 3.21 |
| 1771_s_at | PDGFRB | 5159 | 364.89 | 1167.35 | 3.20 |
| 38378_at | CD53 | 963 | 403.83 | 1289.90 | 3.19 |
| 37398_at | PECAM1 | 5175 | 1307.71 | 4173.89 | 3.19 |
| 37006_at | IGJ | 3512 | 1019.53 | 3251.95 | 3.19 |
| 39827_at | DDIT4 | 54541 | 1375.69 | 4368.48 | 3.18 |
| 36577_at | FERMT2 | 10979 | 603.20 | 1897.76 | 3.15 |
| 32755_at | ACTA2 | 59 | 7137.85 | 22255.46 | 3.12 |
| 32488_at | COL3A1 | 1281 | 4645.82 | 14484.75 | 3.12 |
| 36878_f_at | HLA-DQB1 /// LOC100294318 | 100294318 /// 3119 | 1313.56 | 4089.82 | 3.11 |
| 41839_at | GAS1 | 2619 | 622.17 | 1930.32 | 3.10 |
| 37701_at | RGS2 | 5997 | 1282.10 | 3938.44 | 3.07 |
| 41738_at | CALD1 | 800 | 711.65 | 2179.05 | 3.06 |
| 37716_at | CD200 | 4345 | 411.21 | 1255.66 | 3.05 |
| 41723_s_at | HLA-DRB1 /// HLA-DRB3 /// HLA-DRB4 /// HLA-DRB5 /// HLA-DRB6 /// LOC100294036 /// LOC651845 | 100294036 /// 3123 /// 3125 /// 3126 /// 3127 /// 3128 /// 651845 | 2728.84 | 8275.23 | 3.03 |
| 34091_s_at | VIM | 7431 | 8041.41 | 24355.39 | 3.03 |
| 39945_at | FAP | 2191 | 909.53 | 2754.52 | 3.03 |
| 36149_at | DPYSL3 | 1809 | 759.41 | 2296.35 | 3.02 |
| 39775_at | SERPING1 | 710 | 5707.63 | 17175.70 | 3.01 |
| 36790_at | TPM1 | 7168 | 2478.97 | 7389.63 | 2.98 |
| 36207_at | SEC14L1 | 6397 | 805.01 | 2384.57 | 2.96 |
| 34407_at | RARRES2 | 5919 | 1447.03 | 4268.42 | 2.95 |
| 36979_at | SLC2A3 | 6515 | 1219.10 | 3594.67 | 2.95 |
| 2062_at | IGFBP7 | 3490 | 4190.37 | 12301.83 | 2.94 |
| 38404_at | TGM2 | 7052 | 1233.75 | 3610.64 | 2.93 |
| 38077_at | COL6A3 | 1293 | 5977.85 | 17299.29 | 2.89 |
| 36908_at | MRC1 /// MRC1L1 | 414308 /// 4360 | 491.94 | 1404.48 | 2.85 |
| 38038_at | LUM | 4060 | 3443.02 | 9787.31 | 2.84 |
| 38745_at | LIPA | 3988 | 756.50 | 2148.67 | 2.84 |
| 36861_at | MXRA5 | 25878 | 2904.54 | 8146.52 | 2.80 |
| 40310_at | TLR2 | 7097 | 491.68 | 1374.18 | 2.79 |
| 38767_at | SPRY1 | 10252 | 412.38 | 1141.27 | 2.77 |
| 32128_at | CCL18 | 6362 | 918.90 | 2530.90 | 2.75 |
| 38653_at | PMP22 | 5376 | 2091.70 | 5752.41 | 2.75 |
| 38968_at | SH3BP5 | 9467 | 637.51 | 1723.90 | 2.70 |
| 34303_at | ZCCHC24 | 219654 | 571.36 | 1532.05 | 2.68 |
| 38427_at | COL15A1 | 1306 | 674.28 | 1805.19 | 2.68 |
| 36792_at | TPM1 | 7168 | 3581.29 | 9587.00 | 2.68 |
| 38833_at | HLA-DPA1 | 3113 | 5040.97 | 13063.10 | 2.59 |
| 36638_at | CTGF | 1490 | 3619.86 | 9374.79 | 2.59 |
| 2087_s_at | CDH11 | 1009 | 572.85 | 1477.30 | 2.58 |
| 31672_g_at | RBMS1 | 5937 | 732.87 | 1888.58 | 2.58 |
| 40949_at | DYNC1LI2 | 1783 | 484.10 | 1246.54 | 2.57 |
| 32306_g_at | COL1A2 | 1278 | 10004.56 | 25674.20 | 2.57 |
| 31690_at | GLUD2 | 2747 | 393.39 | 1002.07 | 2.55 |
| 40496_at | C1S | 716 | 4504.86 | 11426.52 | 2.54 |
| 1717_s_at | BIRC3 | 330 | 746.13 | 1879.42 | 2.52 |
| 32249_at | CFH /// CFHR1 | 3075 /// 3078 | 768.86 | 1917.83 | 2.49 |
| 36976_at | CDH11 | 1009 | 478.53 | 1190.51 | 2.49 |
| 37542_at | LHFPL2 | 10184 | 978.27 | 2432.17 | 2.49 |
| 40078_at | PRSS23 | 11098 | 1182.27 | 2933.24 | 2.48 |
| 34375_at | CCL2 | 6347 | 1157.10 | 2860.03 | 2.47 |
| 718_at | HTRA1 | 5654 | 1691.33 | 4170.73 | 2.47 |
| 479_at | DAB2 | 1601 | 457.92 | 1122.47 | 2.45 |
| 33412_at | LGALS1 | 3956 | 7936.72 | 19399.55 | 2.44 |
| 1403_s_at | CCL5 | 6352 | 1325.68 | 3239.81 | 2.44 |
| 31897_at | FILIP1L | 11259 | 428.46 | 1046.53 | 2.44 |
| 38096_f_at | HLA-DPB1 | 3115 | 3088.84 | 7518.44 | 2.43 |
| 1737_s_at | IGFBP4 | 3487 | 1639.34 | 3973.97 | 2.42 |
| 34777_at | ADM | 133 | 2947.16 | 7099.22 | 2.41 |
| 33439_at | SIK1 | 150094 | 2041.01 | 4902.39 | 2.40 |
| 39071_at | ITGAV | 3685 | 717.03 | 1722.07 | 2.40 |
| 32066_g_at | CREM | 1390 | 463.53 | 1111.10 | 2.40 |
| 1104_s_at | HSPA1A /// HSPA1B | 3303 /// 3304 | 5072.73 | 12155.68 | 2.40 |
| 39317_at | CMAH | 8418 | 525.56 | 1255.67 | 2.39 |
| 31432_g_at | FCGRT | 2217 | 1082.12 | 2572.44 | 2.38 |
| 41199_s_at | SFPQ | 6421 | 459.83 | 1091.42 | 2.37 |
| 37759_at | LAPTM5 | 7805 | 1244.69 | 2950.16 | 2.37 |
| 581_at | LAMB1 | 3912 | 687.11 | 1616.78 | 2.35 |
| 36227_at | IL7R | 3575 | 757.30 | 1780.73 | 2.35 |
| 1911_s_at | GADD45A | 1647 | 924.83 | 2166.23 | 2.34 |
| 33813_at | TNFRSF1B | 7133 | 1357.71 | 3161.15 | 2.33 |
| 36773_f_at | HLA-DQB1 | 3119 | 1601.78 | 3728.72 | 2.33 |
| 34268_at | RGS19 | 10287 | 575.10 | 1337.33 | 2.33 |
| 875_g_at | CCL2 | 6347 | 2137.19 | 4938.43 | 2.31 |
| 31692_at | HSPA1A | 3303 | 2055.12 | 4737.04 | 2.30 |
| 37024_at | LITAF | 9516 | 857.29 | 1969.94 | 2.30 |
| 38095_i_at | HLA-DPB1 | 3115 | 7877.02 | 18099.73 | 2.30 |
| 1519_at | ETS2 | 2114 | 1257.45 | 2887.37 | 2.30 |
| 38972_at | KCTD12 | 115207 | 1577.78 | 3600.20 | 2.28 |
| 317_at | LGMN | 5641 | 1803.39 | 4114.96 | 2.28 |
| 35735_at | GBP1 | 2633 | 822.18 | 1866.86 | 2.27 |
| 33352_at | HIST2H2BE | 8349 | 1216.88 | 2759.30 | 2.27 |
| 32593_at | RFTN1 | 23180 | 457.10 | 1034.57 | 2.26 |
| 1867_at | CFLAR | 8837 | 1357.20 | 3062.11 | 2.26 |
| 40848_g_at | MICAL2 | 9645 | 678.27 | 1529.69 | 2.26 |
| 40008_at | CCL11 | 6356 | 678.72 | 1527.90 | 2.25 |
| 38125_at | SERPINE1 | 5054 | 1915.17 | 4298.29 | 2.24 |
| 34859_at | MAGED2 | 10916 | 798.48 | 1789.82 | 2.24 |
| 38422_s_at | FHL2 | 2274 | 977.05 | 2176.00 | 2.23 |
| 32250_at | CFH | 3075 | 606.79 | 1349.84 | 2.22 |
| 33930_at | SCFD1 | 23256 | 561.94 | 1249.93 | 2.22 |
| 514_at | CBLB | 868 | 462.43 | 1025.41 | 2.22 |
| 37344_at | HLA-DMA /// HLA-DMB | 3108 /// 3109 | 1212.58 | 2683.69 | 2.21 |
| 40856_at | SERPINF1 | 5176 | 2721.35 | 5979.74 | 2.20 |
| 943_at | RUNX1 | 861 | 455.76 | 1001.17 | 2.20 |
| 37352_at | SP100 | 6672 | 537.55 | 1179.10 | 2.19 |
| 333_s_at | RBMS1 | 5937 | 1650.73 | 3607.51 | 2.19 |
| 1842_at | DDIT3 /// NR1H3 | 10062 /// 1649 | 1100.57 | 2395.13 | 2.18 |
| 39166_s_at | SERPINH1 | 871 | 3810.87 | 8291.91 | 2.18 |
| 39728_at | IFI30 | 10437 | 11064.71 | 24065.34 | 2.17 |
| 35366_at | NID1 | 4811 | 943.28 | 2043.22 | 2.17 |
| 39333_at | COL4A1 | 1282 | 3645.20 | 7891.87 | 2.17 |
| 33367_s_at | AZIN1 | 51582 | 900.92 | 1943.97 | 2.16 |
| 34800_at | LRIG1 | 26018 | 501.58 | 1080.38 | 2.15 |
| 40448_at | ZFP36 | 7538 | 6264.17 | 13485.90 | 2.15 |
| 36030_at | IFFO1 | 25900 | 479.51 | 1027.31 | 2.14 |
| 33339_g_at | STAT1 | 6772 | 1437.05 | 3072.38 | 2.14 |
| 1850_at | MLH1 | 4292 | 491.22 | 1048.96 | 2.14 |
| 37187_at | CXCL2 | 2920 | 639.53 | 1364.57 | 2.13 |
| 38566_at | COL10A1 | 1300 | 1085.19 | 2306.65 | 2.13 |
| 32587_at | ZFP36L2 | 678 | 2707.58 | 5739.47 | 2.12 |
| 39397_at | NR2F2 | 7026 | 655.01 | 1387.70 | 2.12 |
| 37242_at | FTO | 79068 | 780.73 | 1653.88 | 2.12 |
| 39420_at | DDIT3 /// NR1H3 | 10062 /// 1649 | 1143.59 | 2415.19 | 2.11 |
| 39710_at | C5orf13 | 9315 | 1632.28 | 3439.80 | 2.11 |
| 34342_s_at | SPP1 | 6696 | 7361.19 | 15471.06 | 2.10 |
| 38864_at | NFATC2IP | 84901 | 556.79 | 1170.10 | 2.10 |
| 2092_s_at | SPP1 | 6696 | 3605.64 | 7560.45 | 2.10 |
| 35016_at | CD74 | 972 | 4468.21 | 9357.85 | 2.09 |
| 32818_at | TNC | 3371 | 6114.74 | 12730.97 | 2.08 |
| 34753_at | VAMP7 | 6845 | 898.36 | 1869.24 | 2.08 |
| 1495_at | LTBP1 | 4052 | 1318.71 | 2743.52 | 2.08 |
| 36103_at | CCL3 | 6348 | 802.36 | 1666.72 | 2.08 |
| 32034_at | ZNF217 | 7764 | 493.87 | 1021.06 | 2.07 |
| 39753_at | ITGA5 | 3678 | 1098.97 | 2265.52 | 2.06 |
| 442_at | HSP90B1 | 7184 | 4576.46 | 9420.36 | 2.06 |
| 33143_s_at | SLC16A3 | 9123 | 1136.90 | 2336.89 | 2.06 |
| 36927_at | IFI44L | 10964 | 611.48 | 1255.60 | 2.05 |
| 1815_g_at | TGFBR2 | 7048 | 842.39 | 1718.33 | 2.04 |
| 35303_at | INSIG1 | 3638 | 571.99 | 1166.37 | 2.04 |
| 781_at | RABGGTB | 5876 | 730.67 | 1488.19 | 2.04 |
| 34311_at | GLRX | 2745 | 608.42 | 1238.99 | 2.04 |
| 39376_at | HIPK1 | 204851 | 696.21 | 1416.21 | 2.03 |
| 39409_at | C1R | 715 | 2505.24 | 5076.18 | 2.03 |
| 41191_at | PALLD | 23022 | 5730.64 | 11600.01 | 2.02 |
| 40928_at | WSB1 | 26118 | 1922.82 | 3872.37 | 2.01 |
| 32539_at | COPS8 | 10920 | 637.51 | 1281.98 | 2.01 |
| 37381_g_at | GTF2B | 2959 | 710.44 | 1426.83 | 2.01 |
| 38797_at | SLC39A14 | 23516 | 2721.76 | 5465.58 | 2.01 |
| 31859_at | MMP9 | 4318 | 1130.57 | 2269.35 | 2.01 |
| 33849_at | NAMPT | 10135 | 1290.18 | 2582.08 | 2.00 |
| 41215_s_at | ID2 /// ID2B | 3398 /// 84099 | 1017.58 | 2035.85 | 2.00 |
| 32700_at | GBP2 | 2634 | 616.58 | 1233.51 | 2.00 |
