# Supplementary material for: Artificially Induced Epithelial-Mesenchymal Transition in Surgical Subjects: Its Implications in Clinical and Basic Cancer Research
Source: PLoS One. 2011 Apr 21;6(4):e18196. doi: 10.1371/journal.pone.0018196 (PMC3080870; doi:10.1371/journal.pone.0018196)
Supplement: Table S2 — 716 up-regulated genes in 18 surgically resected esophageal tumors. (DOC) [file pone.0018196.s008.doc]

Table S2. 716 up-regulated genes in 18 surgically resected esophageal tumors

| Probe set ID | Gene symbol | Entrez gene ID | Average signal intensity | | Rethio (Biopsy/Surgical) |
| --- | --- | --- | --- | --- | --- |
|  |  |  | Biopsy | Surgical |  |
| 202992_at | C7 | 730 | 83.14 | 2103.64 | 25.30 |
| 209613_s_at | ADH1B | 125 | 137.47 | 2039.71 | 14.84 |
| 209612_s_at | ADH1B | 125 | 150.08 | 2095.33 | 13.96 |
| 209763_at | CHRDL1 | 91851 | 179.09 | 2299.27 | 12.84 |
| 222722_at | OGN | 4969 | 84.46 | 1056.52 | 12.51 |
| 220276_at | RERGL | 79785 | 23.88 | 253.21 | 10.60 |
| 204940_at | PLN | 5350 | 94.73 | 1003.15 | 10.59 |
| 231773_at | ANGPTL1 | 9068 | 36.06 | 375.09 | 10.40 |
| 223623_at | C2orf40 | 84417 | 92.76 | 950.49 | 10.25 |
| 202274_at | ACTG2 | 72 | 783.11 | 7828.26 | 10.00 |
| 209687_at | CXCL12 | 6387 | 271.81 | 2707.08 | 9.96 |
| 212592_at | IGJ | 3512 | 1589.07 | 15681.64 | 9.87 |
| 225207_at | PDK4 | 5166 | 313.57 | 3079.96 | 9.82 |
| 202988_s_at | RGS1 | 5996 | 447.80 | 4368.70 | 9.76 |
| 218730_s_at | OGN | 4969 | 30.55 | 280.72 | 9.19 |
| 228504_at | SCN7A | 6332 | 87.54 | 731.35 | 8.35 |
| 201496_x_at | MYH11 | 4629 | 314.36 | 2620.29 | 8.34 |
| 210072_at | CCL19 | 6363 | 367.02 | 3052.01 | 8.32 |
| 223395_at | ABI3BP | 25890 | 441.73 | 3610.83 | 8.17 |
| 212713_at | MFAP4 | 4239 | 104.02 | 826.04 | 7.94 |
| 204939_s_at | PLN | 5350 | 314.13 | 2461.62 | 7.84 |
| 206666_at | GZMK | 3003 | 175.48 | 1357.86 | 7.74 |
| 204051_s_at | SFRP4 | 6424 | 206.27 | 1481.07 | 7.18 |
| 205549_at | PCP4 | 5121 | 201.19 | 1441.12 | 7.16 |
| 225720_at | SYNPO2 | 171024 | 515.62 | 3666.61 | 7.11 |
| 206207_at | CLC | 1178 | 130.82 | 927.35 | 7.09 |
| 209101_at | CTGF | 1490 | 617.48 | 4295.96 | 6.96 |
| 207238_s_at | PTPRC | 5788 | 170.02 | 1146.07 | 6.74 |
| 203296_s_at | ATP1A2 | 477 | 105.23 | 705.14 | 6.70 |
| 238481_at | MGP | 4256 | 58.42 | 380.46 | 6.51 |
| 225895_at | SYNPO2 | 171024 | 741.53 | 4798.36 | 6.47 |
| 216834_at | RGS1 | 5996 | 5510.01 | 35097.45 | 6.37 |
| 228592_at | MS4A1 | 931 | 499.77 | 3145.10 | 6.29 |
| 206227_at | CILP | 8483 | 242.54 | 1493.29 | 6.16 |
| 225721_at | SYNPO2 | 171024 | 913.62 | 5615.40 | 6.15 |
| 223122_s_at | SFRP2 | 6423 | 2322.24 | 14245.16 | 6.13 |
| 209201_x_at | CXCR4 | 7852 | 333.76 | 2028.67 | 6.08 |
| 201497_x_at | MYH11 | 4629 | 1872.59 | 11371.62 | 6.07 |
| 232383_at | TFEC | 22797 | 31.05 | 188.24 | 6.06 |
| 209795_at | CD69 | 969 | 424.78 | 2571.04 | 6.05 |
| 228854_at | --- | --- | 205.24 | 1221.04 | 5.95 |
| 215078_at | SOD2 | 6648 | 440.57 | 2604.83 | 5.91 |
| 228202_at | PLN | 5350 | 175.31 | 1008.31 | 5.75 |
| 201858_s_at | SRGN | 5552 | 607.55 | 3449.76 | 5.68 |
| 1558778_s_at | MKL2 | 57496 | 31.23 | 174.17 | 5.58 |
| 205612_at | MMRN1 | 22915 | 79.93 | 443.81 | 5.55 |
| 227662_at | SYNPO2 | 171024 | 932.34 | 5106.95 | 5.48 |
| 224339_s_at | ANGPTL1 | 9068 | 40.56 | 220.20 | 5.43 |
| 205419_at | GPR183 | 1880 | 306.99 | 1635.66 | 5.33 |
| 243797_at | STK17B | 9262 | 57.39 | 305.71 | 5.33 |
| 203305_at | F13A1 | 2162 | 378.48 | 2013.92 | 5.32 |
| 228599_at | MS4A1 | 931 | 80.03 | 424.11 | 5.30 |
| 218087_s_at | SORBS1 | 10580 | 441.44 | 2322.00 | 5.26 |
| 238698_at | CASK | 8573 | 66.98 | 349.77 | 5.22 |
| 230237_at | ADCYAP1 | 116 | 43.18 | 224.83 | 5.21 |
| 239336_at | THBS1 | 7057 | 99.76 | 509.89 | 5.11 |
| 1568760_at | MYH11 | 4629 | 88.31 | 448.14 | 5.07 |
| 210839_s_at | ENPP2 | 5168 | 263.92 | 1335.38 | 5.06 |
| 223121_s_at | SFRP2 | 6423 | 390.81 | 1975.59 | 5.06 |
| 216233_at | CD163 | 9332 | 33.91 | 170.90 | 5.04 |
| 228702_at | FLJ43663 | 378805 | 507.09 | 2545.73 | 5.02 |
| 237206_at | MYOCD | 93649 | 96.86 | 476.51 | 4.92 |
| 219932_at | SLC27A6 | 28965 | 49.20 | 241.71 | 4.91 |
| 219059_s_at | LYVE1 | 10894 | 70.57 | 345.31 | 4.89 |
| 219667_s_at | BANK1 | 55024 | 206.35 | 1002.51 | 4.86 |
| 212187_x_at | PTGDS | 5730 | 514.17 | 2478.15 | 4.82 |
| 203766_s_at | LMOD1 | 25802 | 219.63 | 1055.44 | 4.81 |
| 206030_at | ASPA | 443 | 115.17 | 550.64 | 4.78 |
| 204563_at | SELL | 6402 | 438.96 | 2097.28 | 4.78 |
| 211634_x_at | IGHM /// LOC100133862 | 100133862 /// 3507 | 132.91 | 625.42 | 4.71 |
| 203951_at | CNN1 | 1264 | 343.22 | 1605.33 | 4.68 |
| 202920_at | ANK2 | 287 | 188.67 | 882.41 | 4.68 |
| 211635_x_at | IGH@ /// IGHA1 /// IGHA2 /// IGHD /// IGHG1 /// IGHG3 /// IGHG4 /// IGHM /// IGHV4-31 /// LOC100133862 /// LOC100290528 /// LOC100292483 | 100133862 /// 100290528 /// 100292483 /// 28396 /// 3492 /// 3493 /// 3494 /// 3495 /// 3500 /// 3502 /// 3503 /// 3507 | 106.46 | 494.38 | 4.64 |
| 203543_s_at | KLF9 | 687 | 319.29 | 1470.49 | 4.61 |
| 34210_at | CD52 | 1043 | 409.49 | 1867.83 | 4.56 |
| 204661_at | CD52 | 1043 | 166.63 | 754.81 | 4.53 |
| 217028_at | CXCR4 | 7852 | 3363.58 | 15194.49 | 4.52 |
| 211991_s_at | HLA-DPA1 | 3113 | 1995.01 | 8958.03 | 4.49 |
| 211919_s_at | CXCR4 | 7852 | 379.11 | 1688.11 | 4.45 |
| 216984_x_at | IGL@ /// IGLV2-18 /// IGLV2-23 /// LOC100293440 | 100293440 /// 28813 /// 28814 /// 3535 | 546.74 | 2432.59 | 4.45 |
| 222162_s_at | ADAMTS1 | 9510 | 1020.19 | 4528.34 | 4.44 |
| 212805_at | PRUNE2 | 158471 | 457.54 | 2019.36 | 4.41 |
| 209183_s_at | C10orf10 | 11067 | 671.49 | 2960.68 | 4.41 |
| 204719_at | ABCA8 | 10351 | 416.83 | 1831.61 | 4.39 |
| 202291_s_at | MGP | 4256 | 689.48 | 3027.76 | 4.39 |
| 209541_at | IGF1 | 3479 | 263.59 | 1152.57 | 4.37 |
| 204938_s_at | PLN | 5350 | 55.92 | 244.43 | 4.37 |
| 217767_at | C3 | 718 | 2615.42 | 11412.42 | 4.36 |
| 208450_at | LGALS2 | 3957 | 120.78 | 526.96 | 4.36 |
| 207961_x_at | MYH11 | 4629 | 915.04 | 3992.29 | 4.36 |
| 1553133_at | C9orf72 | 203228 | 187.07 | 812.79 | 4.34 |
| 235412_at | ARHGEF7 | 8874 | 224.22 | 973.57 | 4.34 |
| 219230_at | TMEM100 | 55273 | 182.44 | 792.08 | 4.34 |
| 205422_s_at | ITGBL1 | 9358 | 127.69 | 554.34 | 4.34 |
| 217378_x_at | LOC100130100 /// LOC100291464 | 100130100 /// 100291464 | 689.82 | 2968.56 | 4.30 |
| 225894_at | SYNPO2 | 171024 | 67.97 | 290.16 | 4.27 |
| 204776_at | THBS4 | 7060 | 271.26 | 1156.22 | 4.26 |
| 243296_at | NAMPT | 10135 | 3361.71 | 14266.86 | 4.24 |
| 202157_s_at | CUGBP2 | 10659 | 1202.23 | 5095.58 | 4.24 |
| 202765_s_at | FBN1 | 2200 | 148.39 | 627.57 | 4.23 |
| 216491_x_at | IGHM | 3507 | 171.31 | 723.84 | 4.23 |
| 211796_s_at | TRBC1 | 28639 | 475.38 | 1995.31 | 4.20 |
| 216401_x_at | LOC652493 /// LOC652694 | 652493 /// 652694 | 962.70 | 4035.06 | 4.19 |
| 203645_s_at | CD163 | 9332 | 865.48 | 3626.84 | 4.19 |
| 204803_s_at | RRAD | 6236 | 342.72 | 1429.88 | 4.17 |
| 205544_s_at | CR2 | 1380 | 183.36 | 763.44 | 4.16 |
| 212587_s_at | PTPRC | 5788 | 1335.67 | 5558.86 | 4.16 |
| 212865_s_at | COL14A1 | 7373 | 340.27 | 1411.30 | 4.15 |
| 204273_at | EDNRB | 1910 | 154.37 | 639.80 | 4.14 |
| 208131_s_at | PTGIS | 5740 | 283.74 | 1174.94 | 4.14 |
| 206201_s_at | MEOX2 | 4223 | 48.55 | 200.18 | 4.12 |
| 209621_s_at | PDLIM3 | 27295 | 598.61 | 2463.12 | 4.11 |
| 211571_s_at | VCAN | 1462 | 113.17 | 464.12 | 4.10 |
| 1555745_a_at | LYZ | 4069 | 182.17 | 744.20 | 4.09 |
| 215049_x_at | CD163 | 9332 | 872.25 | 3560.63 | 4.08 |
| 204472_at | GEM | 2669 | 975.78 | 3981.56 | 4.08 |
| 207651_at | GPR171 | 29909 | 216.32 | 877.96 | 4.06 |
| 217281_x_at | IGH@ /// IGHA1 /// IGHA2 /// IGHG1 /// IGHG2 /// IGHG3 /// IGHM /// IGHV4-31 /// LOC100126583 /// LOC100290036 /// LOC100290320 /// LOC100293211 /// LOC652494 | 100126583 /// 100290036 /// 100290320 /// 100293211 /// 28396 /// 3492 /// 3493 /// 3494 /// 3500 /// 3501 /// 3502 /// 3507 /// 652494 | 263.53 | 1068.49 | 4.05 |
| 209829_at | FAM65B | 9750 | 147.39 | 596.68 | 4.05 |
| 1555349_a_at | ITGB2 | 3689 | 199.88 | 806.93 | 4.04 |
| 233813_at | PPP1R16B | 26051 | 202.08 | 813.33 | 4.02 |
| 227260_at | ANKRD10 | 55608 | 1626.19 | 6539.18 | 4.02 |
| 204438_at | MRC1 /// MRC1L1 | 414308 /// 4360 | 759.24 | 3052.48 | 4.02 |
| 234411_x_at | CD44 | 960 | 95.26 | 382.80 | 4.02 |
| 205883_at | ZBTB16 | 7704 | 150.76 | 604.23 | 4.01 |
| 1555938_x_at | VIM | 7431 | 243.29 | 969.26 | 3.98 |
| 201169_s_at | BHLHE40 | 8553 | 235.42 | 936.89 | 3.98 |
| 212588_at | PTPRC | 5788 | 1833.98 | 7298.22 | 3.98 |
| 1555778_a_at | POSTN | 10631 | 741.37 | 2948.01 | 3.98 |
| 205083_at | AOX1 | 316 | 152.09 | 604.77 | 3.98 |
| 238592_at | PDLIM3 | 27295 | 126.01 | 495.83 | 3.93 |
| 205987_at | CD1C | 911 | 166.86 | 655.72 | 3.93 |
| 214319_at | FRY | 10129 | 88.14 | 344.66 | 3.91 |
| 209959_at | NR4A3 | 8013 | 280.43 | 1093.23 | 3.90 |
| 204774_at | EVI2A | 2123 | 658.37 | 2546.38 | 3.87 |
| 210982_s_at | HLA-DRA | 3122 | 4226.75 | 16327.93 | 3.86 |
| 242946_at | CD53 | 963 | 174.40 | 673.16 | 3.86 |
| 218870_at | ARHGAP15 | 55843 | 269.35 | 1039.62 | 3.86 |
| 210084_x_at | TPSAB1 | 7177 | 83.33 | 321.19 | 3.85 |
| 211339_s_at | ITK | 3702 | 286.85 | 1105.14 | 3.85 |
| 208335_s_at | DARC | 2532 | 314.01 | 1202.47 | 3.83 |
| 209447_at | SYNE1 | 23345 | 281.87 | 1074.82 | 3.81 |
| 216510_x_at | IGHA1 /// IGHG1 /// IGHM /// IGHV3-23 /// IGHV4-31 /// LOC100132941 /// LOC100289290 /// LOC100291056 /// LOC100293211 /// LOC100293324 | 100132941 /// 100289290 /// 100291056 /// 100293211 /// 100293324 /// 28396 /// 28442 /// 3493 /// 3500 /// 3507 | 271.11 | 1031.98 | 3.81 |
| 229339_at | MYOCD | 93649 | 123.20 | 467.99 | 3.80 |
| 200974_at | ACTA2 | 59 | 8200.44 | 31115.27 | 3.79 |
| 209821_at | IL33 | 90865 | 301.44 | 1141.24 | 3.79 |
| 216557_x_at | IGHA1 /// IGHD /// IGHG1 /// IGHG3 /// IGHM /// IGHV3-23 /// IGHV4-31 /// LOC100132941 /// LOC100289290 /// LOC100290036 /// LOC100290320 /// LOC100291190 /// LOC100293211 /// LOC100293324 | 100132941 /// 100289290 /// 100290036 /// 100290320 /// 100291190 /// 100293211 /// 100293324 /// 28396 /// 28442 /// 3493 /// 3495 /// 3500 /// 3502 /// 3507 | 341.70 | 1293.13 | 3.78 |
| 219607_s_at | MS4A4A | 51338 | 480.28 | 1811.67 | 3.77 |
| 211645_x_at | --- | --- | 1052.47 | 3962.37 | 3.76 |
| 205547_s_at | TAGLN | 6876 | 1863.88 | 7015.30 | 3.76 |
| 220059_at | STAP1 | 26228 | 117.99 | 442.53 | 3.75 |
| 213160_at | DOCK2 | 1794 | 201.92 | 755.33 | 3.74 |
| 209305_s_at | GADD45B | 4616 | 222.12 | 830.52 | 3.74 |
| 209392_at | ENPP2 | 5168 | 1361.19 | 5083.38 | 3.73 |
| 210299_s_at | FHL1 | 2273 | 270.85 | 1010.86 | 3.73 |
| 211813_x_at | DCN | 1634 | 2903.16 | 10802.84 | 3.72 |
| 202388_at | RGS2 | 5997 | 4579.10 | 16923.48 | 3.70 |
| 208894_at | HLA-DRA | 3122 | 4998.20 | 18472.00 | 3.70 |
| 209189_at | FOS | 2353 | 2565.08 | 9450.70 | 3.68 |
| 213765_at | MFAP5 | 8076 | 163.93 | 603.95 | 3.68 |
| 201041_s_at | DUSP1 | 1843 | 15631.13 | 57209.12 | 3.66 |
| 230847_at | WRNIP1 | 56897 | 469.45 | 1715.01 | 3.65 |
| 215193_x_at | HLA-DRB1 /// HLA-DRB3 /// HLA-DRB4 | 3123 /// 3125 /// 3126 | 4275.84 | 15589.67 | 3.65 |
| 203416_at | CD53 | 963 | 1574.31 | 5701.07 | 3.62 |
| 1555191_a_at | FHL5 | 9457 | 57.32 | 206.99 | 3.61 |
| 202803_s_at | ITGB2 | 3689 | 554.79 | 1999.03 | 3.60 |
| 210356_x_at | MS4A1 | 931 | 173.49 | 621.21 | 3.58 |
| 211734_s_at | FCER1A | 2205 | 145.60 | 521.16 | 3.58 |
| 1555638_a_at | SAMSN1 | 64092 | 217.25 | 776.79 | 3.58 |
| 211908_x_at | IGHG1 | 3500 | 218.34 | 779.93 | 3.57 |
| 228527_s_at | SLC25A37 | 51312 | 57.01 | 203.26 | 3.57 |
| 206211_at | SELE | 6401 | 592.37 | 2105.86 | 3.55 |
| 1552798_a_at | TLR4 | 7099 | 40.28 | 142.98 | 3.55 |
| 203760_s_at | SLA | 6503 | 303.87 | 1077.67 | 3.55 |
| 205624_at | CPA3 | 1359 | 1226.84 | 4336.47 | 3.53 |
| 209994_s_at | ABCB1 /// ABCB4 | 5243 /// 5244 | 135.33 | 476.38 | 3.52 |
| 213714_at | CACNB2 | 783 | 99.87 | 351.43 | 3.52 |
| 1554309_at | EIF4G3 | 8672 | 127.96 | 450.03 | 3.52 |
| 209542_x_at | IGF1 | 3479 | 194.18 | 682.28 | 3.51 |
| 239144_at | B3GAT2 | 135152 | 119.13 | 417.26 | 3.50 |
| 215033_at | TM4SF1 | 4071 | 150.19 | 525.95 | 3.50 |
| 215077_at | --- | --- | 76.89 | 269.18 | 3.50 |
| 238488_at | IPO11 /// LRRC70 | 100130733 /// 51194 | 127.94 | 447.46 | 3.50 |
| 228094_at | AMICA1 | 120425 | 222.49 | 777.43 | 3.49 |
| 228224_at | PRELP | 5549 | 125.58 | 438.74 | 3.49 |
| 207957_s_at | PRKCB | 5579 | 162.24 | 565.35 | 3.48 |
| 211742_s_at | EVI2B | 2124 | 602.01 | 2096.13 | 3.48 |
| 207317_s_at | CASQ2 | 845 | 178.68 | 622.12 | 3.48 |
| 202902_s_at | CTSS | 1520 | 728.92 | 2537.42 | 3.48 |
| 218469_at | GREM1 | 26585 | 2751.59 | 9572.12 | 3.48 |
| 210116_at | SH2D1A | 4068 | 190.19 | 661.52 | 3.48 |
| 1562255_at | SYTL3 | 94120 | 173.82 | 603.09 | 3.47 |
| 214973_x_at | IGHD /// LOC100290059 /// LOC100292999 | 100290059 /// 100292999 /// 3495 | 376.01 | 1300.97 | 3.46 |
| 1554343_a_at | STAP1 | 26228 | 47.98 | 165.61 | 3.45 |
| 210915_x_at | TRBC1 | 28639 | 743.71 | 2562.29 | 3.45 |
| 203649_s_at | PLA2G2A | 5320 | 123.47 | 424.98 | 3.44 |
| 209374_s_at | IGHM | 3507 | 2213.08 | 7590.51 | 3.43 |
| 220330_s_at | SAMSN1 | 64092 | 592.60 | 2024.69 | 3.42 |
| 1552455_at | PRUNE2 | 158471 | 167.34 | 570.95 | 3.41 |
| 206093_x_at | TNXA /// TNXB | 7146 /// 7148 | 123.77 | 422.22 | 3.41 |
| 1558662_s_at | BANK1 | 55024 | 227.11 | 774.72 | 3.41 |
| 211302_s_at | PDE4B | 5142 | 104.44 | 356.08 | 3.41 |
| 218232_at | C1QA | 712 | 391.07 | 1332.25 | 3.41 |
| 208763_s_at | TSC22D3 | 1831 | 1897.32 | 6436.53 | 3.39 |
| 232629_at | PROK2 | 60675 | 90.98 | 308.51 | 3.39 |
| 206682_at | CLEC10A | 10462 | 194.52 | 659.20 | 3.39 |
| 204052_s_at | SFRP4 | 6424 | 247.46 | 837.74 | 3.39 |
| 1557166_at | PDCD4 | 27250 | 41.71 | 140.92 | 3.38 |
| 205158_at | RNASE4 | 6038 | 190.04 | 641.25 | 3.37 |
| 215379_x_at | IGL@ /// IGLV1-44 | 28823 /// 3535 | 14697.89 | 49578.73 | 3.37 |
| 1554779_s_at | PHLDB2 | 90102 | 119.21 | 401.98 | 3.37 |
| 236778_at | --- | --- | 485.02 | 1633.54 | 3.37 |
| 207802_at | CRISP3 | 10321 | 954.58 | 3213.10 | 3.37 |
| 234103_at | KCNT2 | 343450 | 87.69 | 295.11 | 3.37 |
| 227762_at | --- | --- | 284.51 | 956.19 | 3.36 |
| 209606_at | CYTIP | 9595 | 737.84 | 2477.03 | 3.36 |
| 217148_x_at | LOC100293440 | 100293440 | 1337.32 | 4483.18 | 3.35 |
| 206978_at | CCR2 | 729230 | 186.86 | 624.13 | 3.34 |
| 228055_at | NAPSB | 256236 | 144.23 | 480.46 | 3.33 |
| 219279_at | DOCK10 | 55619 | 299.85 | 998.62 | 3.33 |
| 204894_s_at | AOC3 | 8639 | 439.59 | 1463.02 | 3.33 |
| 210034_s_at | RPL5 | 6125 | 230.74 | 767.12 | 3.32 |
| 211748_x_at | PTGDS | 5730 | 834.57 | 2771.37 | 3.32 |
| 213451_x_at | TNXA /// TNXB | 7146 /// 7148 | 131.16 | 435.07 | 3.32 |
| 215382_x_at | TPSAB1 | 7177 | 107.41 | 355.86 | 3.31 |
| 209083_at | CORO1A | 11151 | 393.62 | 1304.02 | 3.31 |
| 209312_x_at | HLA-DRB1 /// HLA-DRB4 /// HLA-DRB5 | 3123 /// 3126 /// 3127 | 8137.84 | 26943.79 | 3.31 |
| 235777_at | ANKRD44 | 91526 | 88.24 | 292.11 | 3.31 |
| 209199_s_at | MEF2C | 4208 | 733.82 | 2426.99 | 3.31 |
| 1555884_at | PSMD6 | 9861 | 52.71 | 173.86 | 3.30 |
| 200795_at | SPARCL1 | 8404 | 7765.44 | 25553.59 | 3.29 |
| 1562637_at | SAMD12 | 401474 | 58.14 | 190.79 | 3.28 |
| 213416_at | ITGA4 | 3676 | 408.93 | 1341.31 | 3.28 |
| 210954_s_at | TSC22D2 | 9819 | 51.69 | 169.02 | 3.27 |
| 210432_s_at | SCN3A | 6328 | 68.92 | 225.28 | 3.27 |
| 203799_at | CD302 | 9936 | 467.43 | 1525.27 | 3.26 |
| 211794_at | FYB | 2533 | 160.43 | 523.25 | 3.26 |
| 1569599_at | SAMSN1 | 64092 | 50.39 | 164.06 | 3.26 |
| 201289_at | CYR61 | 3491 | 3983.55 | 12955.99 | 3.25 |
| 205242_at | CXCL13 | 10563 | 944.12 | 3058.47 | 3.24 |
| 226103_at | NEXN | 91624 | 436.55 | 1408.41 | 3.23 |
| 208791_at | CLU | 1191 | 992.43 | 3193.93 | 3.22 |
| 228471_at | ANKRD44 | 91526 | 467.02 | 1501.42 | 3.21 |
| 230494_at | SLC20A1 | 6574 | 1413.68 | 4542.97 | 3.21 |
| 201058_s_at | MYL9 | 10398 | 819.42 | 2631.18 | 3.21 |
| 205132_at | ACTC1 | 70 | 268.88 | 862.46 | 3.21 |
| 204236_at | FLI1 | 2313 | 343.35 | 1096.27 | 3.19 |
| 242767_at | LMCD1 | 29995 | 138.86 | 441.32 | 3.18 |
| 206134_at | ADAMDEC1 | 27299 | 532.72 | 1692.63 | 3.18 |
| 204118_at | CD48 | 962 | 463.84 | 1471.38 | 3.17 |
| 206898_at | CDH19 | 28513 | 162.14 | 511.28 | 3.15 |
| 1556498_at | RPL5 | 6125 | 56.44 | 177.74 | 3.15 |
| 203697_at | FRZB | 2487 | 328.07 | 1032.66 | 3.15 |
| 209924_at | CCL18 | 6362 | 1025.74 | 3222.34 | 3.14 |
| 211896_s_at | DCN | 1634 | 4461.78 | 14014.86 | 3.14 |
| 205681_at | BCL2A1 | 597 | 246.96 | 775.72 | 3.14 |
| 228186_s_at | RSPO3 | 84870 | 305.39 | 955.05 | 3.13 |
| 216333_x_at | TNXA /// TNXB | 7146 /// 7148 | 130.11 | 405.72 | 3.12 |
| 210656_at | EED | 8726 | 246.41 | 765.93 | 3.11 |
| 204931_at | TCF21 | 6943 | 126.97 | 394.31 | 3.11 |
| 215051_x_at | AIF1 | 199 | 656.46 | 2038.09 | 3.10 |
| 202672_s_at | ATF3 | 467 | 2027.41 | 6271.52 | 3.09 |
| 209823_x_at | HLA-DQB1 | 3119 | 944.13 | 2916.84 | 3.09 |
| 1554602_at | RBM8A | 9939 | 234.14 | 722.17 | 3.08 |
| 215925_s_at | CD72 | 971 | 68.43 | 211.01 | 3.08 |
| 221031_s_at | APOLD1 | 81575 | 1143.42 | 3517.21 | 3.08 |
| 205997_at | ADAM28 | 10863 | 324.59 | 997.72 | 3.07 |
| 224357_s_at | MS4A4A | 51338 | 118.93 | 364.92 | 3.07 |
| 208792_s_at | CLU | 1191 | 1790.85 | 5469.19 | 3.05 |
| 230130_at | --- | --- | 450.24 | 1371.79 | 3.05 |
| 201137_s_at | HLA-DPB1 | 3115 | 5212.22 | 15869.76 | 3.04 |
| 227510_x_at | MALAT1 | 378938 | 1465.78 | 4454.66 | 3.04 |
| 204951_at | RHOH | 399 | 255.72 | 775.62 | 3.03 |
| 203471_s_at | PLEK | 5341 | 414.99 | 1257.96 | 3.03 |
| 223501_at | TNFSF13B | 10673 | 894.46 | 2710.41 | 3.03 |
| 203698_s_at | FRZB | 2487 | 285.41 | 864.17 | 3.03 |
| 202746_at | ITM2A | 9452 | 459.81 | 1390.13 | 3.02 |
| 1554929_at | QSK | 23387 | 51.88 | 156.47 | 3.02 |
| 224356_x_at | MS4A6A | 64231 | 839.45 | 2527.56 | 3.01 |
| 209457_at | DUSP5 | 1847 | 796.81 | 2398.21 | 3.01 |
| 215646_s_at | VCAN | 1462 | 170.58 | 513.31 | 3.01 |
| 1554741_s_at | FGF7 /// KGFLP1 /// KGFLP2 | 2252 /// 387628 /// 654466 | 432.15 | 1300.01 | 3.01 |
| 222043_at | CLU | 1191 | 962.19 | 2893.19 | 3.01 |
| 243140_at | ACTA2 | 59 | 248.92 | 747.87 | 3.00 |
| 209683_at | FAM49A | 81553 | 91.07 | 272.56 | 2.99 |
| 1556990_at | PERP | 64065 | 62.63 | 187.09 | 2.99 |
| 235944_at | HMCN1 | 83872 | 434.56 | 1294.99 | 2.98 |
| 206398_s_at | CD19 | 930 | 115.89 | 345.11 | 2.98 |
| 223280_x_at | MS4A6A | 64231 | 880.04 | 2620.23 | 2.98 |
| 212671_s_at | HLA-DQA1 /// HLA-DQA2 /// LOC100294224 /// LOC100294317 | 100294224 /// 100294317 /// 3117 /// 3118 | 3164.21 | 9381.23 | 2.96 |
| 232304_at | PELI1 | 57162 | 990.39 | 2933.55 | 2.96 |
| 225242_s_at | CCDC80 | 151887 | 1695.99 | 5023.37 | 2.96 |
| 226841_at | MPEG1 | 219972 | 665.36 | 1968.30 | 2.96 |
| 212827_at | IGHM | 3507 | 277.32 | 818.96 | 2.95 |
| 210401_at | P2RX1 | 5023 | 96.97 | 286.27 | 2.95 |
| 206637_at | P2RY14 | 9934 | 534.21 | 1573.35 | 2.95 |
| 223343_at | MS4A7 | 58475 | 1038.49 | 3054.69 | 2.94 |
| 206214_at | PLA2G7 | 7941 | 533.87 | 1569.34 | 2.94 |
| 1555843_at | HNRNPM | 4670 | 640.98 | 1883.81 | 2.94 |
| 205226_at | PDGFRL | 5157 | 136.23 | 399.87 | 2.94 |
| 206392_s_at | RARRES1 | 5918 | 194.59 | 570.61 | 2.93 |
| 226641_at | --- | --- | 808.85 | 2365.12 | 2.92 |
| 208885_at | LCP1 | 3936 | 608.74 | 1775.34 | 2.92 |
| 239328_at | --- | --- | 143.37 | 417.98 | 2.92 |
| 201721_s_at | LAPTM5 | 7805 | 3006.16 | 8729.15 | 2.90 |
| 211577_s_at | IGF1 | 3479 | 174.93 | 506.61 | 2.90 |
| 219243_at | GIMAP4 | 55303 | 250.02 | 723.94 | 2.90 |
| 215784_at | CD1E | 913 | 56.44 | 163.40 | 2.90 |
| 205922_at | VNN2 | 8875 | 354.07 | 1025.02 | 2.89 |
| 222142_at | CYLD | 1540 | 280.90 | 812.25 | 2.89 |
| 206049_at | SELP | 6403 | 440.21 | 1271.24 | 2.89 |
| 209960_at | HGF | 3082 | 185.12 | 534.54 | 2.89 |
| 206715_at | TFEC | 22797 | 230.01 | 663.81 | 2.89 |
| 204834_at | FGL2 | 10875 | 1007.79 | 2904.79 | 2.88 |
| 204959_at | MNDA | 4332 | 570.66 | 1644.42 | 2.88 |
| 210322_x_at | UTY | 7404 | 164.01 | 472.03 | 2.88 |
| 204890_s_at | LCK | 3932 | 181.27 | 521.57 | 2.88 |
| 224126_at | SLC10A7 | 84068 | 52.11 | 149.90 | 2.88 |
| 219574_at | 1-Mar | 55016 | 228.41 | 656.63 | 2.87 |
| 228071_at | GIMAP7 | 168537 | 423.67 | 1217.81 | 2.87 |
| 211470_s_at | SULT1C2 | 6819 | 128.89 | 370.19 | 2.87 |
| 214470_at | KLRB1 | 3820 | 196.66 | 564.74 | 2.87 |
| 211798_x_at | IGLJ3 | 28831 | 738.21 | 2118.29 | 2.87 |
| 206932_at | CH25H | 9023 | 509.72 | 1461.27 | 2.87 |
| 211324_s_at | RGPD5 /// RGPD7 /// RGPD8 | 652919 /// 727851 /// 84220 | 47.57 | 136.16 | 2.86 |
| 203761_at | SLA | 6503 | 798.98 | 2285.71 | 2.86 |
| 60084_at | CYLD | 1540 | 227.09 | 647.83 | 2.85 |
| 205804_s_at | TRAF3IP3 | 80342 | 169.44 | 482.69 | 2.85 |
| 206496_at | FMO3 | 2328 | 213.99 | 608.81 | 2.85 |
| 210764_s_at | CYR61 | 3491 | 503.49 | 1431.10 | 2.84 |
| 208306_x_at | --- | 730415 | 11425.80 | 32453.94 | 2.84 |
| 208983_s_at | PECAM1 | 5175 | 194.46 | 552.11 | 2.84 |
| 240369_at | TTC7A | 57217 | 111.92 | 317.57 | 2.84 |
| 239027_at | DOCK8 | 81704 | 94.26 | 267.12 | 2.83 |
| 201531_at | ZFP36 | 7538 | 2837.43 | 8030.32 | 2.83 |
| 218468_s_at | GREM1 | 26585 | 2489.55 | 7040.05 | 2.83 |
| 214927_at | ITGBL1 | 9358 | 286.03 | 808.71 | 2.83 |
| 205051_s_at | KIT | 3815 | 454.43 | 1283.52 | 2.82 |
| 235778_s_at | ANKRD44 | 91526 | 72.56 | 204.48 | 2.82 |
| 227529_s_at | AKAP12 | 9590 | 166.61 | 469.37 | 2.82 |
| 211881_x_at | IGLJ3 | 28831 | 562.54 | 1584.01 | 2.82 |
| 205407_at | RECK | 8434 | 551.91 | 1553.64 | 2.82 |
| 212998_x_at | HLA-DQB1 /// LOC100294318 | 100294318 /// 3119 | 1879.41 | 5272.35 | 2.81 |
| 223344_s_at | MS4A7 | 58475 | 181.77 | 509.77 | 2.80 |
| 227346_at | IKZF1 | 10320 | 846.10 | 2368.59 | 2.80 |
| 231124_x_at | LY9 | 4063 | 237.14 | 663.43 | 2.80 |
| 203473_at | SLCO2B1 | 11309 | 530.01 | 1480.77 | 2.79 |
| 217480_x_at | LOC100287723 /// LOC642424 /// LOC642838 | 100287723 /// 642424 /// 642838 | 921.88 | 2575.12 | 2.79 |
| 220532_s_at | TMEM176B | 28959 | 740.38 | 2066.56 | 2.79 |
| 207574_s_at | GADD45B | 4616 | 1819.02 | 5070.99 | 2.79 |
| 203708_at | PDE4B | 5142 | 993.75 | 2769.89 | 2.79 |
| 213071_at | DPT | 1805 | 209.89 | 585.02 | 2.79 |
| 221019_s_at | COLEC12 | 81035 | 325.63 | 906.76 | 2.78 |
| 230422_at | FPR3 | 2359 | 318.41 | 885.87 | 2.78 |
| 1555812_a_at | ARHGDIB | 397 | 528.42 | 1469.36 | 2.78 |
| 202340_x_at | NR4A1 | 3164 | 423.05 | 1176.27 | 2.78 |
| 201893_x_at | DCN | 1634 | 9236.23 | 25671.06 | 2.78 |
| 240052_at | ITPR1 | 3708 | 167.91 | 466.67 | 2.78 |
| 223987_at | CHRDL2 | 25884 | 104.76 | 290.53 | 2.77 |
| 219213_at | JAM2 | 58494 | 343.07 | 950.59 | 2.77 |
| 214340_at | ALOX12P2 | 245 | 153.67 | 425.32 | 2.77 |
| 202436_s_at | CYP1B1 | 1545 | 565.73 | 1565.49 | 2.77 |
| 1553856_s_at | P2RY10 | 27334 | 95.12 | 262.91 | 2.76 |
| 1555724_s_at | TAGLN | 6876 | 308.50 | 850.65 | 2.76 |
| 204982_at | GIT2 | 9815 | 233.60 | 643.68 | 2.76 |
| 234308_at | TUBGCP6 | 85378 | 121.88 | 335.14 | 2.75 |
| 231093_at | FCRL3 | 115352 | 338.91 | 931.19 | 2.75 |
| 222915_s_at | BANK1 | 55024 | 104.04 | 285.72 | 2.75 |
| 219666_at | MS4A6A | 64231 | 1014.35 | 2783.43 | 2.74 |
| 205159_at | CSF2RB | 1439 | 1608.49 | 4410.40 | 2.74 |
| 219471_at | C13orf18 | 80183 | 299.68 | 821.49 | 2.74 |
| 225782_at | MSRB3 | 253827 | 1961.75 | 5349.67 | 2.73 |
| 223925_s_at | MTPN | 136319 | 50.41 | 137.29 | 2.72 |
| 203088_at | FBLN5 | 10516 | 186.65 | 507.44 | 2.72 |
| 209656_s_at | TMEM47 | 83604 | 1271.87 | 3453.33 | 2.72 |
| 201540_at | FHL1 | 2273 | 6168.34 | 16723.84 | 2.71 |
| 231911_at | ERMN | 57471 | 72.81 | 196.97 | 2.71 |
| 210889_s_at | FCGR2B | 2213 | 216.12 | 584.42 | 2.70 |
| 210538_s_at | BIRC3 | 330 | 1342.79 | 3630.91 | 2.70 |
| 206618_at | IL18R1 | 8809 | 155.84 | 421.30 | 2.70 |
| 207547_s_at | FAM107A | 11170 | 184.38 | 498.42 | 2.70 |
| 227058_at | C13orf33 | 84935 | 261.37 | 706.08 | 2.70 |
| 240413_at | PYHIN1 | 149628 | 96.18 | 259.80 | 2.70 |
| 230550_at | MS4A6A | 64231 | 331.94 | 893.09 | 2.69 |
| 229127_at | --- | --- | 250.37 | 672.92 | 2.69 |
| 243352_at | ALPK1 | 80216 | 309.87 | 830.52 | 2.68 |
| 238699_s_at | CASK | 8573 | 112.24 | 300.69 | 2.68 |
| 1557257_at | BCL10 | 8915 | 272.02 | 727.77 | 2.68 |
| 203381_s_at | APOE | 348 | 389.14 | 1039.42 | 2.67 |
| 1556474_a_at | FLJ38379 | 285097 | 102.28 | 272.13 | 2.66 |
| 222486_s_at | ADAMTS1 | 9510 | 95.72 | 254.58 | 2.66 |
| 205782_at | FGF7 | 2252 | 88.65 | 235.74 | 2.66 |
| 218805_at | GIMAP5 | 55340 | 482.01 | 1280.30 | 2.66 |
| 217022_s_at | IGH@ /// IGHA1 /// IGHA2 /// LOC100126583 | 100126583 /// 3492 /// 3493 /// 3494 | 21553.34 | 57237.64 | 2.66 |
| 1555728_a_at | MS4A4A | 51338 | 192.21 | 509.47 | 2.65 |
| 215967_s_at | LY9 | 4063 | 86.23 | 228.45 | 2.65 |
| 204606_at | CCL21 | 6366 | 149.09 | 393.69 | 2.64 |
| 209436_at | SPON1 | 10418 | 881.14 | 2323.44 | 2.64 |
| 1554547_at | FAM13C | 220965 | 510.74 | 1343.18 | 2.63 |
| 213566_at | RNASE6 | 6039 | 522.13 | 1371.09 | 2.63 |
| 205119_s_at | FPR1 | 2357 | 440.67 | 1155.77 | 2.62 |
| 233288_at | ATR | 545 | 89.04 | 233.44 | 2.62 |
| 202664_at | WIPF1 | 7456 | 1697.26 | 4439.66 | 2.62 |
| 242734_x_at | GALT | 2592 | 54.72 | 143.09 | 2.61 |
| 242814_at | SERPINB9 | 5272 | 1112.90 | 2910.07 | 2.61 |
| 231274_s_at | --- | --- | 1001.28 | 2617.44 | 2.61 |
| 228410_at | GAB3 | 139716 | 157.24 | 410.38 | 2.61 |
| 213436_at | CNR1 | 1268 | 154.20 | 401.89 | 2.61 |
| 209685_s_at | PRKCB | 5579 | 423.64 | 1103.62 | 2.61 |
| 224341_x_at | TLR4 | 7099 | 128.06 | 333.58 | 2.60 |
| 201957_at | PPP1R12B | 4660 | 635.69 | 1655.08 | 2.60 |
| 209210_s_at | FERMT2 | 10979 | 3479.78 | 9054.26 | 2.60 |
| 214366_s_at | ALOX5 | 240 | 80.85 | 210.35 | 2.60 |
| 229725_at | ACSL6 | 23305 | 46.54 | 121.01 | 2.60 |
| 209901_x_at | AIF1 | 199 | 265.78 | 689.55 | 2.59 |
| 208077_at | C9orf38 | 29044 | 93.21 | 241.41 | 2.59 |
| 241616_at | --- | --- | 62.82 | 162.47 | 2.59 |
| 227645_at | PIK3R5 | 23533 | 156.04 | 403.53 | 2.59 |
| 232568_at | MGC24103 | 158295 | 420.50 | 1086.03 | 2.58 |
| 1554676_at | SRGN | 5552 | 72.21 | 186.11 | 2.58 |
| 202957_at | HCLS1 | 3059 | 1433.38 | 3684.56 | 2.57 |
| 214060_at | SSBP1 | 6742 | 271.94 | 699.02 | 2.57 |
| 1557080_s_at | ITGBL1 | 9358 | 207.75 | 533.89 | 2.57 |
| 1565754_x_at | FGD2 | 221472 | 205.98 | 528.67 | 2.57 |
| 205504_at | BTK | 695 | 157.71 | 404.16 | 2.56 |
| 1554921_a_at | SCEL | 8796 | 222.92 | 571.29 | 2.56 |
| 205357_s_at | AGTR1 | 185 | 129.74 | 332.47 | 2.56 |
| 201525_at | APOD | 347 | 286.16 | 733.09 | 2.56 |
| 230836_at | ST8SIA4 | 7903 | 400.58 | 1025.96 | 2.56 |
| 230925_at | APBB1IP | 54518 | 569.21 | 1456.65 | 2.56 |
| 202283_at | SERPINF1 | 5176 | 1529.41 | 3909.44 | 2.56 |
| 215602_at | FGD2 | 221472 | 102.93 | 262.96 | 2.55 |
| 242292_at | CXorf50B | 653687 | 91.10 | 232.28 | 2.55 |
| 217757_at | A2M | 2 | 5020.32 | 12760.67 | 2.54 |
| 220301_at | CCDC102B | 79839 | 182.87 | 464.65 | 2.54 |
| 213375_s_at | N4BP2L1 | 90634 | 1041.61 | 2646.27 | 2.54 |
| 227530_at | AKAP12 | 9590 | 732.13 | 1859.66 | 2.54 |
| 242784_at | --- | --- | 280.36 | 711.70 | 2.54 |
| 210815_s_at | CALCRL | 10203 | 71.67 | 181.64 | 2.53 |
| 228728_at | C7orf58 | 79974 | 464.57 | 1175.42 | 2.53 |
| 205237_at | FCN1 | 2219 | 164.60 | 415.74 | 2.53 |
| 225442_at | DDR2 | 4921 | 909.14 | 2295.46 | 2.52 |
| 222513_s_at | SORBS1 | 10580 | 461.61 | 1162.22 | 2.52 |
| 232267_at | GPR133 | 283383 | 137.84 | 346.79 | 2.52 |
| 1569003_at | TMEM49 | 81671 | 3689.51 | 9277.85 | 2.51 |
| 236341_at | CTLA4 | 1493 | 476.13 | 1197.08 | 2.51 |
| 209894_at | LEPR | 3953 | 605.85 | 1521.53 | 2.51 |
| 232204_at | EBF1 | 1879 | 211.03 | 527.38 | 2.50 |
| 201852_x_at | COL3A1 | 1281 | 12876.81 | 32083.70 | 2.49 |
| 223997_at | FNIP1 | 96459 | 92.03 | 229.17 | 2.49 |
| 209200_at | MEF2C | 4208 | 728.86 | 1813.47 | 2.49 |
| 1555103_s_at | FGF7 | 2252 | 38.13 | 94.86 | 2.49 |
| 205270_s_at | LCP2 | 3937 | 308.26 | 766.69 | 2.49 |
| 205857_at | SLC18A2 | 6571 | 138.75 | 344.82 | 2.49 |
| 213975_s_at | LYZ | 4069 | 7758.44 | 19259.02 | 2.48 |
| 210198_s_at | PLP1 | 5354 | 99.85 | 247.66 | 2.48 |
| 233011_at | ANXA1 | 301 | 171.37 | 424.76 | 2.48 |
| 203868_s_at | VCAM1 | 7412 | 2244.13 | 5548.46 | 2.47 |
| 235593_at | ZEB2 | 9839 | 140.39 | 346.93 | 2.47 |
| 204642_at | S1PR1 | 1901 | 331.16 | 818.16 | 2.47 |
| 1568768_s_at | LOC100302650 /// RBKS | 100302650 /// 64080 | 119.67 | 295.54 | 2.47 |
| 214511_x_at | FCGR1B | 2210 | 187.33 | 462.56 | 2.47 |
| 219371_s_at | KLF2 | 10365 | 695.06 | 1715.85 | 2.47 |
| 224856_at | FKBP5 | 2289 | 752.92 | 1856.83 | 2.47 |
| 219971_at | IL21R | 50615 | 57.12 | 140.68 | 2.46 |
| 227368_at | --- | --- | 814.88 | 2005.20 | 2.46 |
| 238189_at | SIN3A | 25942 | 65.36 | 160.55 | 2.46 |
| 1555476_at | IREB2 | 3658 | 139.09 | 341.04 | 2.45 |
| 219778_at | ZFPM2 | 23414 | 245.71 | 602.16 | 2.45 |
| 205269_at | LCP2 | 3937 | 513.38 | 1257.66 | 2.45 |
| 1558972_s_at | THEMIS | 387357 | 118.16 | 288.30 | 2.44 |
| 212192_at | KCTD12 | 115207 | 4186.77 | 10211.01 | 2.44 |
| 207777_s_at | SP140 | 11262 | 166.47 | 405.98 | 2.44 |
| 223809_at | RGS18 | 64407 | 177.95 | 433.69 | 2.44 |
| 202766_s_at | FBN1 | 2200 | 5290.74 | 12881.46 | 2.43 |
| 1568765_at | SERPINE1 | 5054 | 146.18 | 355.84 | 2.43 |
| 1569225_a_at | SCML4 | 256380 | 88.17 | 214.44 | 2.43 |
| 216033_s_at | FYN | 2534 | 261.18 | 634.26 | 2.43 |
| 204749_at | NAP1L3 | 4675 | 141.44 | 342.44 | 2.42 |
| 1569701_at | PER3 | 8863 | 116.15 | 281.03 | 2.42 |
| 1552315_at | GIMAP1 | 170575 | 135.23 | 326.92 | 2.42 |
| 219191_s_at | BIN2 | 51411 | 104.32 | 252.14 | 2.42 |
| 201743_at | CD14 | 929 | 1198.74 | 2895.03 | 2.42 |
| 210972_x_at | TRA@ /// TRAC /// TRAJ17 /// TRAV20 | 28663 /// 28738 /// 28755 /// 6955 | 222.84 | 537.43 | 2.41 |
| 203603_s_at | ZEB2 | 9839 | 312.03 | 751.93 | 2.41 |
| 211654_x_at | HLA-DQB1 | 3119 | 1641.98 | 3952.46 | 2.41 |
| 1556323_at | CUGBP2 | 10659 | 122.62 | 294.96 | 2.41 |
| 235306_at | GIMAP8 | 155038 | 605.42 | 1455.73 | 2.40 |
| 1562031_at | JAK2 | 3717 | 75.47 | 181.19 | 2.40 |
| 207697_x_at | LILRB2 | 10288 | 180.81 | 433.65 | 2.40 |
| 217208_s_at | DLG1 | 1739 | 348.17 | 834.82 | 2.40 |
| 211336_x_at | LILRB1 | 10859 | 133.94 | 321.08 | 2.40 |
| 212758_s_at | ZEB1 | 6935 | 574.84 | 1376.69 | 2.39 |
| 204482_at | CLDN5 | 7122 | 465.37 | 1114.04 | 2.39 |
| 209993_at | ABCB1 | 5243 | 125.61 | 300.67 | 2.39 |
| 1554899_s_at | FCER1G | 2207 | 113.71 | 272.06 | 2.39 |
| 202995_s_at | FBLN1 | 2192 | 317.81 | 760.06 | 2.39 |
| 207474_at | SNRK | 54861 | 304.55 | 728.31 | 2.39 |
| 231947_at | MYCT1 | 80177 | 193.23 | 461.64 | 2.39 |
| 205859_at | LY86 | 9450 | 484.54 | 1157.60 | 2.39 |
| 243864_at | CCDC80 | 151887 | 135.64 | 323.83 | 2.39 |
| 211795_s_at | FYB | 2533 | 1056.78 | 2521.11 | 2.39 |
| 211395_x_at | FCGR2C | 9103 | 186.92 | 445.79 | 2.38 |
| 215236_s_at | PICALM | 8301 | 130.06 | 310.16 | 2.38 |
| 207190_at | ZZEF1 | 23140 | 99.91 | 238.03 | 2.38 |
| 217633_at | URB1 | 9875 | 114.31 | 271.96 | 2.38 |
| 242726_at | --- | --- | 129.87 | 308.63 | 2.38 |
| 223922_x_at | MS4A6A | 64231 | 630.61 | 1495.27 | 2.37 |
| 1552822_at | TMX3 | 54495 | 103.56 | 245.04 | 2.37 |
| 228772_at | HNMT | 3176 | 217.87 | 515.18 | 2.36 |
| 216542_x_at | IGHA1 /// IGHG1 /// IGHM /// IGHV3-23 /// LOC100290059 /// LOC100290293 /// LOC100292999 /// LOC100293211 | 100290059 /// 100290293 /// 100292999 /// 100293211 /// 28442 /// 3493 /// 3500 /// 3507 | 390.53 | 922.17 | 2.36 |
| 205116_at | LAMA2 | 3908 | 396.59 | 936.24 | 2.36 |
| 236244_at | HNRNPU | 3192 | 216.25 | 510.40 | 2.36 |
| 1557352_at | SQLE | 6713 | 630.54 | 1488.17 | 2.36 |
| 235061_at | PPM1K | 152926 | 810.59 | 1911.54 | 2.36 |
| 227874_at | EMCN | 51705 | 408.81 | 963.59 | 2.36 |
| 239364_at | --- | --- | 201.55 | 474.64 | 2.35 |
| 226769_at | FIBIN | 387758 | 663.41 | 1561.73 | 2.35 |
| 210751_s_at | RGN | 9104 | 140.95 | 331.72 | 2.35 |
| 204007_at | FCGR3B | 2215 | 816.66 | 1916.49 | 2.35 |
| 212188_at | KCTD12 | 115207 | 1867.57 | 4379.39 | 2.34 |
| 204112_s_at | HNMT | 3176 | 870.32 | 2039.09 | 2.34 |
| 201150_s_at | TIMP3 | 7078 | 2380.73 | 5577.63 | 2.34 |
| 1569021_at | PIK3C2A | 5286 | 75.61 | 176.97 | 2.34 |
| 232068_s_at | TLR4 | 7099 | 60.93 | 142.56 | 2.34 |
| 206407_s_at | CCL13 | 6357 | 217.61 | 508.93 | 2.34 |
| 1552807_a_at | SIGLEC10 /// SIGLEC12 | 89790 /// 89858 | 394.97 | 921.02 | 2.33 |
| 235735_at | --- | --- | 220.96 | 514.96 | 2.33 |
| 240572_s_at | LOC374443 | 374443 | 245.38 | 571.78 | 2.33 |
| 1563228_x_at | SLC38A10 | 124565 | 109.03 | 253.94 | 2.33 |
| 219728_at | MYOT | 9499 | 68.62 | 159.70 | 2.33 |
| 210765_at | CSE1L | 1434 | 296.69 | 690.36 | 2.33 |
| 204670_x_at | HLA-DRB1 /// HLA-DRB4 | 3123 /// 3126 | 9021.18 | 20967.59 | 2.32 |
| 226906_s_at | ARHGAP9 | 64333 | 132.35 | 306.73 | 2.32 |
| 210571_s_at | CMAH | 8418 | 83.26 | 192.92 | 2.32 |
| 203923_s_at | CYBB | 1536 | 354.36 | 820.48 | 2.32 |
| 235504_at | GREM2 | 64388 | 65.13 | 150.73 | 2.31 |
| 211317_s_at | CFLAR | 8837 | 204.01 | 471.55 | 2.31 |
| 220518_at | ABI3BP | 25890 | 83.67 | 193.32 | 2.31 |
| 206481_s_at | LDB2 | 9079 | 700.80 | 1618.19 | 2.31 |
| 228194_s_at | SORCS1 | 114815 | 48.48 | 111.93 | 2.31 |
| 204787_at | VSIG4 | 11326 | 424.21 | 979.27 | 2.31 |
| 223620_at | GPR34 | 2857 | 364.26 | 840.68 | 2.31 |
| 241643_at | TLK1 | 9874 | 21.32 | 49.19 | 2.31 |
| 235385_at | --- | --- | 172.23 | 396.47 | 2.30 |
| 230011_at | MEI1 | 150365 | 193.47 | 444.84 | 2.30 |
| 211459_at | --- | --- | 72.95 | 167.54 | 2.30 |
| 1568840_at | DCAF13 | 25879 | 83.64 | 191.71 | 2.29 |
| 224964_s_at | GNG2 | 54331 | 1206.70 | 2762.95 | 2.29 |
| 1570042_a_at | ADAM9 | 8754 | 196.39 | 449.09 | 2.29 |
| 240573_at | LOC374443 | 374443 | 109.37 | 249.87 | 2.28 |
| 210895_s_at | CD86 | 942 | 257.33 | 587.06 | 2.28 |
| 206828_at | TXK | 7294 | 66.16 | 150.75 | 2.28 |
| 205695_at | SDS | 10993 | 154.32 | 351.52 | 2.28 |
| 240338_at | --- | --- | 169.33 | 385.48 | 2.28 |
| 211220_s_at | HSF2 | 3298 | 57.59 | 131.04 | 2.28 |
| 240405_at | --- | --- | 155.88 | 354.62 | 2.27 |
| 1553134_s_at | C9orf72 | 203228 | 51.92 | 118.03 | 2.27 |
| 203680_at | PRKAR2B | 5577 | 523.97 | 1190.90 | 2.27 |
| 1569941_at | --- | --- | 34.14 | 77.59 | 2.27 |
| 201235_s_at | BTG2 | 7832 | 166.98 | 379.42 | 2.27 |
| 216236_s_at | SLC2A14 /// SLC2A3 | 144195 /// 6515 | 298.87 | 678.88 | 2.27 |
| 202158_s_at | CUGBP2 | 10659 | 399.78 | 907.51 | 2.27 |
| 1552508_at | KCNE4 | 23704 | 41.37 | 93.87 | 2.27 |
| 206785_s_at | KLRC1 /// KLRC2 | 3821 /// 3822 | 307.71 | 697.42 | 2.27 |
| 211902_x_at | TRA@ | 6955 | 155.87 | 352.66 | 2.26 |
| 1557719_at | PIKFYVE | 200576 | 136.04 | 307.13 | 2.26 |
| 224560_at | TIMP2 | 7077 | 1996.95 | 4503.46 | 2.26 |
| 237756_at | PHOSPHO2 | 493911 | 81.29 | 183.29 | 2.25 |
| 228030_at | --- | --- | 3452.81 | 7783.35 | 2.25 |
| 244599_at | --- | --- | 250.79 | 565.28 | 2.25 |
| 219505_at | CECR1 | 51816 | 915.18 | 2056.04 | 2.25 |
| 230543_at | USP9X | 8239 | 155.54 | 349.35 | 2.25 |
| 232617_at | CTSS | 1520 | 1970.60 | 4423.37 | 2.24 |
| 237379_at | PHRF1 | 57661 | 102.21 | 229.39 | 2.24 |
| 231747_at | CYSLTR1 | 10800 | 259.05 | 580.82 | 2.24 |
| 235372_at | FCRLA | 84824 | 241.78 | 542.09 | 2.24 |
| 238257_at | MLLT10 | 8028 | 50.13 | 112.22 | 2.24 |
| 214615_at | P2RY10 | 27334 | 92.18 | 206.32 | 2.24 |
| 1569864_at | SERAC1 | 84947 | 107.14 | 239.79 | 2.24 |
| 214761_at | ZNF423 | 23090 | 411.99 | 921.98 | 2.24 |
| 240570_at | INADL | 10207 | 134.58 | 300.81 | 2.24 |
| 212915_at | PDZRN3 | 23024 | 889.03 | 1985.97 | 2.23 |
| 232031_s_at | KIAA1632 | 57724 | 130.74 | 292.06 | 2.23 |
| 213888_s_at | TRAF3IP3 | 80342 | 597.04 | 1333.62 | 2.23 |
| 211286_x_at | CSF2RA | 1438 | 74.01 | 165.22 | 2.23 |
| 242776_at | ZCCHC6 | 79670 | 117.66 | 262.29 | 2.23 |
| 228047_at | SNORA72 | 26775 | 198.53 | 441.79 | 2.23 |
| 202238_s_at | NNMT | 4837 | 1121.25 | 2493.62 | 2.22 |
| 205038_at | IKZF1 | 10320 | 114.78 | 255.19 | 2.22 |
| 218162_at | OLFML3 | 56944 | 609.39 | 1354.18 | 2.22 |
| 1558199_at | FN1 | 2335 | 245.91 | 546.42 | 2.22 |
| 207001_x_at | TSC22D3 | 1831 | 87.87 | 195.12 | 2.22 |
| 213994_s_at | SPON1 | 10418 | 865.58 | 1921.64 | 2.22 |
| 204083_s_at | TPM2 | 7169 | 2338.87 | 5185.04 | 2.22 |
| 227646_at | EBF1 | 1879 | 1026.19 | 2273.94 | 2.22 |
| 214533_at | CMA1 | 1215 | 57.07 | 126.44 | 2.22 |
| 1569652_at | MLLT3 | 4300 | 327.76 | 726.12 | 2.22 |
| 202018_s_at | LTF | 4057 | 1892.22 | 4181.49 | 2.21 |
| 231067_s_at | --- | --- | 159.94 | 353.26 | 2.21 |
| 219947_at | CLEC4A | 50856 | 785.11 | 1733.94 | 2.21 |
| 1554660_a_at | C1orf71 | 163882 | 109.89 | 242.52 | 2.21 |
| 1554966_a_at | FILIP1L | 11259 | 556.16 | 1225.96 | 2.20 |
| 201617_x_at | CALD1 | 800 | 823.80 | 1814.61 | 2.20 |
| 204674_at | LRMP | 4033 | 678.62 | 1494.36 | 2.20 |
| 203474_at | IQGAP2 | 10788 | 525.79 | 1157.58 | 2.20 |
| 230983_at | FAM129C | 199786 | 157.72 | 347.17 | 2.20 |
| 220704_at | IKZF1 | 10320 | 86.37 | 190.04 | 2.20 |
| 205653_at | CTSG | 1511 | 211.83 | 465.89 | 2.20 |
| 222067_x_at | HIST1H2BD | 3017 | 1260.74 | 2770.88 | 2.20 |
| 209708_at | MOXD1 | 26002 | 832.25 | 1828.47 | 2.20 |
| 216840_s_at | LAMA2 | 3908 | 530.01 | 1162.43 | 2.19 |
| 228167_at | KLHL6 | 89857 | 462.56 | 1014.00 | 2.19 |
| 203561_at | FCGR2A | 2212 | 650.04 | 1422.81 | 2.19 |
| 215058_at | DENND5B | 160518 | 57.63 | 125.94 | 2.19 |
| 1558996_at | FOXP1 | 27086 | 187.54 | 409.71 | 2.18 |
| 211824_x_at | NLRP1 | 22861 | 121.22 | 264.64 | 2.18 |
| 209583_s_at | CD200 | 4345 | 435.68 | 950.82 | 2.18 |
| 202878_s_at | CD93 | 22918 | 2561.47 | 5584.13 | 2.18 |
| 226436_at | RASSF4 | 83937 | 573.17 | 1246.88 | 2.18 |
| 243495_s_at | --- | --- | 1045.82 | 2274.48 | 2.17 |
| 240528_s_at | EXOC4 | 60412 | 169.29 | 367.77 | 2.17 |
| 242277_at | --- | --- | 943.81 | 2049.48 | 2.17 |
| 206117_at | TPM1 | 7168 | 181.28 | 393.59 | 2.17 |
| 201785_at | RNASE1 | 6035 | 824.21 | 1785.63 | 2.17 |
| 208442_s_at | ATM | 472 | 263.92 | 571.76 | 2.17 |
| 229218_at | COL1A2 | 1278 | 3710.67 | 8028.75 | 2.16 |
| 209324_s_at | RGS16 | 6004 | 191.04 | 413.14 | 2.16 |
| 226878_at | HLA-DOA | 3111 | 437.42 | 944.98 | 2.16 |
| 232461_at | AHI1 | 54806 | 84.90 | 183.21 | 2.16 |
| 35974_at | LRMP | 4033 | 418.09 | 901.95 | 2.16 |
| 230389_at | FNBP1 | 23048 | 1823.01 | 3928.37 | 2.15 |
| 209243_s_at | PEG3 /// ZIM2 | 23619 /// 5178 | 44.27 | 95.24 | 2.15 |
| 226244_at | CLEC14A | 161198 | 189.17 | 406.98 | 2.15 |
| 232687_at | --- | --- | 307.41 | 661.08 | 2.15 |
| 202499_s_at | SLC2A3 | 6515 | 1214.91 | 2610.28 | 2.15 |
| 207691_x_at | ENTPD1 | 953 | 392.39 | 842.15 | 2.15 |
| 242943_at | ST8SIA4 | 7903 | 151.76 | 325.68 | 2.15 |
| 238478_at | BNC2 | 54796 | 349.63 | 749.58 | 2.14 |
| 204072_s_at | FRY | 10129 | 622.29 | 1332.60 | 2.14 |
| 224833_at | ETS1 | 2113 | 5956.26 | 12753.47 | 2.14 |
| 203029_s_at | PTPRN2 | 5799 | 201.67 | 431.23 | 2.14 |
| 208146_s_at | CPVL | 54504 | 1448.13 | 3089.73 | 2.13 |
| 213095_x_at | AIF1 | 199 | 443.81 | 945.86 | 2.13 |
| 206701_x_at | EDNRB | 1910 | 122.78 | 261.51 | 2.13 |
| 225798_at | JAZF1 | 221895 | 703.48 | 1496.91 | 2.13 |
| 222934_s_at | CLEC4E | 26253 | 126.89 | 269.89 | 2.13 |
| 209474_s_at | ENTPD1 | 953 | 235.74 | 500.12 | 2.12 |
| 211466_at | NFIB | 4781 | 82.62 | 175.00 | 2.12 |
| 229487_at | EBF1 | 1879 | 347.13 | 734.73 | 2.12 |
| 234801_s_at | ACSS1 | 84532 | 156.61 | 331.20 | 2.11 |
| 205885_s_at | ITGA4 | 3676 | 69.59 | 147.01 | 2.11 |
| 230645_at | FRMD3 | 257019 | 137.42 | 289.91 | 2.11 |
| 210365_at | RUNX1 | 861 | 220.54 | 464.87 | 2.11 |
| 232762_at | KIAA1217 | 56243 | 659.36 | 1388.39 | 2.11 |
| 232599_at | EXOC6 | 54536 | 46.77 | 98.37 | 2.10 |
| 220399_at | NCRNA00115 | 79854 | 289.89 | 609.34 | 2.10 |
| 240757_at | CLASP1 | 23332 | 228.60 | 479.94 | 2.10 |
| 221760_at | MAN1A1 | 4121 | 2687.75 | 5637.25 | 2.10 |
| 210387_at | HIST1H2BG | 8339 | 732.28 | 1535.26 | 2.10 |
| 202887_s_at | DDIT4 | 54541 | 8775.89 | 18398.51 | 2.10 |
| 223502_s_at | TNFSF13B | 10673 | 1189.29 | 2491.69 | 2.10 |
| 242388_x_at | --- | --- | 143.59 | 300.58 | 2.09 |
| 230516_at | C7orf30 | 115416 | 425.18 | 889.71 | 2.09 |
| 226039_at | MGAT4A | 11320 | 1115.09 | 2325.81 | 2.09 |
| 230276_at | FAM49A | 81553 | 266.72 | 556.28 | 2.09 |
| 238611_at | --- | --- | 669.15 | 1394.79 | 2.08 |
| 216248_s_at | NR4A2 | 4929 | 885.75 | 1845.67 | 2.08 |
| 203470_s_at | PLEK | 5341 | 382.73 | 797.43 | 2.08 |
| 215199_at | CALD1 | 800 | 215.38 | 448.66 | 2.08 |
| 1552480_s_at | PTPRC | 5788 | 30.96 | 64.41 | 2.08 |
| 235040_at | RUNDC1 | 146923 | 94.00 | 195.54 | 2.08 |
| 214701_s_at | FN1 | 2335 | 146.16 | 303.80 | 2.08 |
| 209182_s_at | C10orf10 | 11067 | 122.39 | 254.33 | 2.08 |
| 202497_x_at | SLC2A3 | 6515 | 625.75 | 1298.56 | 2.08 |
| 227266_s_at | FYB | 2533 | 964.53 | 1996.24 | 2.07 |
| 244029_at | LOC100131043 | 100131043 | 269.17 | 556.89 | 2.07 |
| 202833_s_at | SERPINA1 | 5265 | 277.14 | 572.81 | 2.07 |
| 221541_at | CRISPLD2 | 83716 | 2647.69 | 5469.98 | 2.07 |
| 232864_s_at | AFF4 | 27125 | 127.92 | 264.23 | 2.07 |
| 220146_at | TLR7 | 51284 | 184.18 | 380.30 | 2.06 |
| 211095_at | NF1 | 4763 | 113.11 | 233.48 | 2.06 |
| 1565898_at | METT5D1 | 196074 | 67.19 | 138.67 | 2.06 |
| 219747_at | C4orf31 | 79625 | 202.33 | 417.23 | 2.06 |
| 1555411_a_at | CCNL1 | 57018 | 1989.26 | 4101.03 | 2.06 |
| 1553861_at | TCP11L2 | 255394 | 88.71 | 182.79 | 2.06 |
| 211557_x_at | SLCO2B1 | 11309 | 64.33 | 132.53 | 2.06 |
| 210222_s_at | RTN1 | 6252 | 120.92 | 248.98 | 2.06 |
| 201279_s_at | DAB2 | 1601 | 380.95 | 783.56 | 2.06 |
| 202450_s_at | CTSK | 1513 | 5921.43 | 12166.35 | 2.05 |
| 203914_x_at | HPGD | 3248 | 818.79 | 1682.20 | 2.05 |
| 222846_at | RAB8B | 51762 | 199.56 | 408.98 | 2.05 |
| 207075_at | NLRP3 | 114548 | 150.24 | 307.88 | 2.05 |
| 227297_at | ITGA9 | 3680 | 552.21 | 1130.33 | 2.05 |
| 238429_at | TMEM71 | 137835 | 366.31 | 749.39 | 2.05 |
| 1554493_s_at | THADA | 63892 | 123.26 | 251.53 | 2.04 |
| 203167_at | TIMP2 | 7077 | 460.08 | 938.39 | 2.04 |
| 219179_at | DACT1 | 51339 | 337.53 | 688.27 | 2.04 |
| 1556420_s_at | YPEL2 | 388403 | 230.44 | 468.92 | 2.03 |
| 207067_s_at | HDC | 3067 | 217.16 | 441.73 | 2.03 |
| 204446_s_at | ALOX5 | 240 | 1280.64 | 2603.20 | 2.03 |
| 222528_s_at | SLC25A37 | 51312 | 168.77 | 342.93 | 2.03 |
| 1563229_at | DLEU2 | 8847 | 107.11 | 217.54 | 2.03 |
| 210439_at | ICOS | 29851 | 353.71 | 718.11 | 2.03 |
| 205668_at | LY75 | 4065 | 1161.79 | 2358.64 | 2.03 |
| 228426_at | CLEC2D | 29121 | 59.24 | 120.16 | 2.03 |
| 1555397_at | MYO1D | 4642 | 67.47 | 136.83 | 2.03 |
| 227817_at | PRKCB | 5579 | 175.71 | 355.91 | 2.03 |
| 203854_at | CFI | 3426 | 1092.01 | 2211.19 | 2.02 |
| 201110_s_at | THBS1 | 7057 | 2117.33 | 4283.37 | 2.02 |
| 226814_at | ADAMTS9 | 56999 | 366.16 | 739.67 | 2.02 |
| 220416_at | ATP8B4 | 79895 | 96.19 | 194.27 | 2.02 |
| 229723_at | TAGAP | 117289 | 564.46 | 1136.42 | 2.01 |
| 232898_at | DAB2 | 1601 | 137.12 | 275.45 | 2.01 |
| 242280_x_at | CPEB4 | 80315 | 417.92 | 838.75 | 2.01 |
| 224166_at | SLC25A2 | 83884 | 79.42 | 159.23 | 2.00 |
