# Supplementary material for: Artificially Induced Epithelial-Mesenchymal Transition in Surgical Subjects: Its Implications in Clinical and Basic Cancer Research
Source: PLoS One. 2011 Apr 21;6(4):e18196. doi: 10.1371/journal.pone.0018196 (PMC3080870; doi:10.1371/journal.pone.0018196)
Supplement: Table S3 — Clinicopathological information of biopsy samples from different cases with esophageal squamous cell carcinoma. (DOC) [file pone.0018196.s009.doc]

Table S3. Clinicopathological information of biopsy samples from different cases with esophageal squamous cell carcinoma

| No. | Tissue | Age | Sex | TNM  stage | Survival term (days) |
| --- | --- | --- | --- | --- | --- |
| A-1-1 | Tumor | 56 | M | III | >2171 |
| A-1-2 | Tumor | 61 | M | IV | >2233 |
| A-1-3 | Tumor | 68 | M | III | >2012 |
| A-1-4 | Tumor | 48 | M | IIA | >1891 |
| A-1-5 | Tumor | 55 | M | IIA | >2076 |
| A-1-6 | Tumor | 54 | M | IIB | >1857 |
| A-1-7 | Tumor | 67 | M | IIA | >1901 |
| A-1-8 | Tumor | 68 | M | III | >1733 |
| A-1-9 | Tumor | 50 | M | III | >1527 |
| A-1-10 | Tumor | 65 | M | III | >1367 |
| A-1-11 | Tumor | 72 | M | IIA | >1366 |
| A-1-12 | Tumor | 64 | F | III | >1346 |
| A-1-13 | Tumor | 58 | F | III | >1233 |
| A-1-14 | Tumor | 72 | M | IIA | >1136 |
| D-1-1 | Tumor | 72 | F | III | 379 |
| D-1-2 | Tumor | 60 | M | III | 251 |
| D-1-3 | Tumor | 61 | M | IV | 227 |
| D-1-4 | Tumor | 49 | M | IV | 239 |
| D-1-5 | Tumor | 53 | M | IIA | 364 |
| D-1-6 | Tumor | 50 | M | IV | 226 |
| D-1-7 | Tumor | 55 | M | IIB | 280 |
| D-1-8 | Tumor | 70 | M | IV | 299 |
| D-1-9 | Tumor | 72 | M | IV | 156 |
| D-1-10 | Tumor | 75 | M | IIA | 309 |
| D-1-11 | Tumor | 67 | M | III | 236 |
| A-2-1 | Tumor | 71 | M | IIA | >912 |
| A-2-2 | Tumor | 70 | M | III | >1018 |
| A-2-3 | Tumor | 44 | M | IIA | >919 |
| A-2-4 | Tumor | 55 | M | IIA | >959 |
| D-2-3 | Tumor | 61 | M | III | 968 |
| D-2-1 | Tumor | 71 | M | III | 664 |
| D-2-2 | Tumor | 64 | M | IIB | 299 |
| D-2-4 | Tumor | 59 | F | IIA | 521 |
| D-2-5 | Tumor | 66 | M | IIA | 1018 |
| D-2-6 | Tumor | 57 | M | IIA | 1114 |
| A-1-4N | Normal | 48 | M | IIA | >1891 |
| A-1-12N | Normal | 64 | F | III | >1346 |
| A-1-13N | Normal | 58 | F | III | >1233 |
| A-1-14N | Normal | 72 | M | IIA | >1136 |
| A-2-4N | Normal | 55 | M | IIA | >959 |
