# Supplementary material for: Artificially Induced Epithelial-Mesenchymal Transition in Surgical Subjects: Its Implications in Clinical and Basic Cancer Research
Source: PLoS One. 2011 Apr 21;6(4):e18196. doi: 10.1371/journal.pone.0018196 (PMC3080870; doi:10.1371/journal.pone.0018196)
Supplement: Table S4 — Clinicopathological information of surgical samples from different cases with esophageal squamous cell carcinoma. (DOC) [file pone.0018196.s010.doc]

Table S4. Clinicopathological information of surgical samples from different cases with esophageal squamous cell carcinoma

| No. | Tissue | Age | Sex | TNM  stage | Survival term (days) |
| --- | --- | --- | --- | --- | --- |
| O-1-1 | Tumor | 57 | M | III | 47 |
| O-1-2 | Tumor | 71 | F | IIA | >1544 |
| O-1-3 | Tumor | 70 | F | IIA | 1194 |
| O-1-4 | Tumor | 57 | M | IIA | >2304 |
| O-1-5 | Tumor | 67 | F | IIA | >2425 |
| O-1-6 | Tumor | 66 | F | IIA | >1589 |
| O-1-7 | Tumor | 61 | M | III | 66 |
| O-1-8 | Tumor | 63 | M | IIA | >2369 |
| O-1-9 | Tumor | 49 | M | IIA | >1588 |
| O-1-10 | Tumor | 76 | M | IIA | 351 |
| O-1-11 | Tumor | 58 | M | III | 1136 |
| O-1-12 | Tumor | 64 | M | IIA | >1569 |
| O-1-13 | Tumor | 60 | M | IIA | 2536 |
| O-1-14 | Tumor | 56 | M | I | >2665 |
| O-1-15 | Tumor | 69 | M | IIA | >2755 |
| O-1-16 | Tumor | 66 | M | I | >2782 |
| O-1-17 | Tumor | 56 | M | I | >2665 |
| O-2-1 | Tumor | 71 | M | III | >2204 |
| O-2-2 | Tumor | 66 | M | III | 514 |
| O-2-3 | Tumor | 55 | M | III | 423 |
| O-2-4 | Tumor | 57 | M | III | >1977 |
| O-2-5 | Tumor | 50 | M | III | >2020 |
| O-2-6 | Tumor | 75 | M | IV | >1683 |
| O-3-1 | Tumor | 67 | M | III | 475 |
| O-3-2 | Tumor | 64 | M | IV | 472 |
| O-3-3 | Tumor | 48 | M | IVA | 309 |
| O-3-4 | Tumor | 76 | M | III | 95 |
| O-3-5 | Tumor | 76 | M | IV | 659 |
| O-3-6 | Tumor | 62 | M | IV | 40 |
| O-3-7 | Tumor | 68 | M | IV | 432 |
| O-3-8 | Tumor | 48 | M | IV | 640 |
| O-3-9 | Tumor | 64 | M | IV | 1439 |
| O-3-10 | Tumor | 67 | M | IV | 772 |
| O-3-11 | Tumor | 67 | M | III | 856 |
| O-3-12 | Tumor | 68 | M | IV | 622 |
| O-3-13 | Tumor | 66 | M | II | 291 |
| O-3-14 | Tumor | 44 | M | III | 204 |
| O-3-15 | Tumor | 76 | M | IV | 247 |
| O-3-16 | Tumor | 53 | M | III | 429 |
| O-3-17 | Tumor | 58 | M | IV | 116 |
| O-3-18 | Tumor | 58 | M | III | 195 |
| O-3-19 | Tumor | 75 | M | III | 1045 |
| O-3-20 | Tumor | 59 | M | III | 316 |
| O-3-21 | Tumor | 54 | M | IV | 297 |
| O-3-22 | Tumor | 62 | M | III | 476 |
| O-3-23 | Tumor | 66 | M | III | 419 |
| O-3-24 | Tumor | 73 | M | III | 502 |
| O-3-25 | Tumor | 55 | F | IV | 510 |
| O-3-26 | Tumor | 55 | M | IV | 46 |
| O-3-27 | Tumor | 60 | M | III | 168 |
| O-3-28 | Tumor | 52 | M | IV | 68 |
| O-3-29 | Tumor | 53 | M | IV | 929 |
| O-3-30 | Tumor | 60 | M | IV | 579 |
| O-4-1 | Tumor | 62 | M | IV | 616 |
| O-4-2 | Tumor | 68 | M | III | 433 |
| O-4-3 | Tumor | 60 | M | IV | 38 |
| O-4-4 | Tumor | 72 | M | IV | 157 |
| O-4-5 | Tumor | 54 | M | IV | 114 |
| O-4-6 | Tumor | 62 | M | IV | 156 |
| O-4-7 | Tumor | Unknown | Unknown | Unknown | 624 |
| O-4-8 | Tumor | 77 | M | III | 24 |
| O-4-9 | Tumor | 70 | F | IV | >1567 |
| O-4-10 | Tumor | 80 | M | III | 770 |
| O-4-11 | Tumor | 64 | M | IV | 748 |
| O-4-12 | Tumor | 62 | M | III | 372 |
| O-4-13 | Tumor | 66 | M | I | >2782 |
| O-3-6N | Normal | 47 | M | III | 152 |
| O-233N | Normal | Unknown | F | IIA | >2434 |
| O-1-15N | Normal | 69 | M | IIA | >2755 |
| O-438N | Normal | 63 | M | IIA | >2687 |
| O-1-13N | Normal | 60 | M | IIA | 2536 |
