# Supplementary material for: Artificially Induced Epithelial-Mesenchymal Transition in Surgical Subjects: Its Implications in Clinical and Basic Cancer Research
Source: PLoS One. 2011 Apr 21;6(4):e18196. doi: 10.1371/journal.pone.0018196 (PMC3080870; doi:10.1371/journal.pone.0018196)
Supplement: Table S5 — Clinicopathological information of biopsy and surgical samples from different cases with esophageal squamous cell carcinoma. (DOC) [file pone.0018196.s011.doc]

Table S5. Clinicopathological information of biopsy and surgical samples from identical cases with esophageal squamous cell carcinoma

| No. | Tissue | Age | Sex | TNM  stage | Survival term (days) |
| --- | --- | --- | --- | --- | --- |
| BPY/OPE015 | Tumor | 60 | M | III | >1645 |
| BPY/OPE030 | Tumor | 62 | M | IIB | >1438 |
| BPY/OPE041 | Tumor | 51 | M | IIA | >1478 |
| BPY/OPE049 | Tumor | 56 | M | IIB | 1093 |
| BPY/OPE078 | Tumor | 65 | M | IIA | Unknown |
| BPY/OPE094 | Tumor | 55 | M | IIB | >1620 |
| BPY/OPE113 | Tumor | 66 | F | III | 355 |
| BPY/OPE120 | Tumor | 70 | M | IIB | >1270 |
| BPY/OPE124 | Tumor | 71 | M | III | >239 |
| BPY/OPE127 | Tumor | 65 | M | Unknown | Unknown |
| BPY/OPE128 | Tumor | 71 | M | III | >1080 |
| BPY/OPE129 | Tumor | 57 | M | IIA | >1118 |
| BPY/OPE131 | Tumor | 62 | M | III | >1062 |
| BPY/OPE136 | Tumor | 61 | M | IV | 212 |
| BPY/OPE143 | Tumor | 72 | F | IIA | >1140 |
| BPY/OPE145 | Tumor | 69 | M | III | >1174 |
| BPY/OPE148 | Tumor | 73 | F | IIA | >1143 |
| BPY/OPE162 | Tumor | 64 | M | III | >1096 |
